# Cross-Tissue Concordance of a T1D Blood-Derived B-Cell Signature with Hashimoto Thyroiditis Tissue: Exploratory Spatial Immune-Topic Analysis

**DOI:** 10.3390/ijms27219698

**Published:** 2026-10-29

**Authors:** Eun Young Joo, Sang Hun Lee

**Affiliations:** 1 Department of Pediatrics, Inha University College of Medicine, Inha University Hospital, Incheon 22332, Republic of Korea; freegana@inha.ac.kr; 2 Department of Biomedical Sciences, College of Medicine, BK21 FOUR Program in Biomedical Science & Engineering, Inha University, Incheon 22332, Republic of Korea

**Keywords:** type 1 diabetes, Hashimoto thyroiditis, B-cell-associated program, 14-gene frozen signature, cross-tissue transcriptomics, immune-cell heterogeneity, exploratory spatial transcriptomics, APC/HLA-II

## Abstract

Type 1 diabetes (T1D) and Hashimoto thyroiditis (HT) share autoimmune features, but cross-tissue concordance of B-cell transcriptional programs remains uncertain. Using 24 retained cohort–pathway effect estimates, we recalculated T1D blood summary-level meta-analysis statistics and separately report the previously generated frozen 14-gene HT thyroid association. The T1D meta-analysis yielded Hedges’ g = 0.51 (95% CI 0.18–0.85; q = 0.022; I^2^ = 0%). In HRA001684 (16 HT, 50 non-HT), the frozen score was associated with HT (HC3-adjusted β = 2.65, 95% CI 2.37–2.94; permutation *P* = 2 × 10^−5^). Single-cell data (6512 cells; three HT and three immune-checkpoint-inhibitor-associated thyroiditis libraries) lacked healthy controls and were analyzed descriptively. In five spatial sections, the frozen-signature contrast was nonsignificant (Δ = 0.459; exact *P* = 0.10; matched-gene *P* = 0.085), with no preferential correspondence to B/plasma-cell topics (reported *P* = 0.90). Independently inferred thyroid immune topics showed regional variation but did not localize the frozen score. These reported findings are compatible with partial T1D blood–HT thyroid transcriptional concordance but do not establish systemic HT activation, a shared cell-intrinsic program, or validated spatial localization. In the reported sensitivity analysis, joint B-cell/plasma-cell marker adjustment attenuated the HT association by approximately 90%, with the fully adjusted confidence interval including zero. Comparisons with equal weighting and other B-cell reference signatures did not establish a statistically significant advantage of the T1D-derived weights. The public GitHub workflow verifies summary-level pooling of retained effects only; source-to-result reconstruction of the HT bulk, spatial, single-cell, and marker/weight analyses has not been established.

## 1. Introduction

Type 1 diabetes (T1D) and Hashimoto thyroiditis (HT), a form of autoimmune thyroid disease, commonly coexist [1,2,3]. Although they affect different endocrine tissues, both disorders involve impaired immune tolerance, HLA-associated susceptibility, lymphocyte activation, antigen presentation, and interferon-related inflammation [2,3,4]. These similarities raise the possibility of partially shared immune programs. However, whether a transcriptional signature derived from T1D peripheral blood is associated with HT thyroid tissue remains unclear. Detecting such a signature in thyroid tissue would not itself establish its activation in HT peripheral blood.

B cells contribute to both diseases but operate in distinct tissue environments. In T1D, they produce autoantibodies, present β-cell antigens, and interact with autoreactive T cells [5,6]. HT is characterized by intrathyroidal B-cell infiltration and germinal-center formation, including thyroid antigen-specific B cells that participate in local antigen presentation [7,8]. Single-cell studies have identified expanded B-cell and germinal-center-associated populations in HT thyroid tissue [9]. Thus, a thyroid B-cell-associated signal may reflect local immune infiltration or activation rather than a circulating program.

B-cell activity also intersects with interferon, JAK–STAT, and antigen-presentation pathways. In T1D, interferon–JAK–STAT signaling contributes to HLA class I hyperexpression in pancreatic β cells and may facilitate recognition by cytotoxic T cells [4]. In HT, lymphocyte–thyrocyte–stromal interactions contribute to inflammatory injury [10]. These findings place B-cell-associated transcription within broader, tissue-dependent immune networks.

Bulk transcriptomes cannot identify the cells generating a signal or its anatomical distribution. Single-cell datasets can characterize immune-lineage composition, while spatial transcriptomics can describe regional patterns; neither establishes localization of a transferred blood-derived signature without direct statistical tests and suitable controls. Moreover, a thyroid spatial topic inferred independently from RNA expression is not equivalent to the prespecified T1D signature. Prior spatial studies have documented heterogeneous thyroid follicular, stromal, vascular, and immune compartments [11].

Previous cross-disease comparisons have primarily examined circulating immune cells or proteins rather than a fixed B-cell transcriptional score. In a mass-cytometry study of autoimmune endocrine disorders, Magnusson et al. reported disease-specific immune-cell alterations, including increased circulating plasmablasts in HT [12]. A subsequent plasma-protein analysis found higher CDCP1 in T1D and HT and elevated SLAMF1 in long-standing T1D and in HT [13]. These results establish immunological overlap but do not demonstrate preservation of an identical B-cell transcriptional program.

Transcriptomic comparisons have also identified shared target-organ responses. Yi et al. analyzed bulk and single-cell/single-nucleus data across six autoimmune or inflammatory conditions, including T1D pancreatic islets and HT thyroid tissue, and reported convergent inflammatory pathways while examining FGFR1 as a candidate tissue-protective target [14]. Other pancreatic [15,16] and thyroid studies [9,10] have described disease-specific cellular contexts. It remains unclear whether a prespecified T1D blood-derived B-cell score is associated with HT thyroid status. We addressed this question by applying a frozen 14-gene weighted signature to an independent HT bulk cohort and evaluating permutation and matched-gene null distributions; this design does not test systemic B-cell activation in HT.

Whether the blood-derived score occurs in pancreatic-islet immune cells is a separate, untested question. Histopathological studies have documented variable CD20-positive B-cell infiltration in T1D [15,16], and human islet single-cell atlases offer potential transcriptomic resources [17,18]. Direct testing would require adequate B-cell recovery, gene detection, and donor-level comparisons; anatomical localization would require spatial or histological evidence.

We assessed the consistency of B-cell-associated effects across T1D blood cohorts, transferred a fixed 14-gene score to HT bulk thyroid tissue, and characterized complementary single-cell and spatial immune features. The original 15-gene module and expanded 17-gene candidate set are described only for historical provenance; the former is not retained as a separate quantitative HT finding. The current cross-tissue analysis centers on the frozen 14-gene weighted score. The earlier and fixed HT bulk scores were evaluated in the same HRA001684 cohort and do not constitute independent patient-cohort replication. Single-cell and spatial analyses were interpreted descriptively, without inferring circulating HT activation or localization of the transferred signature.

## 2. Results

### 2.1. T1D Peripheral-Blood B-Cell Program and Exploratory Thyroid Immune Context

**Figure 1:**
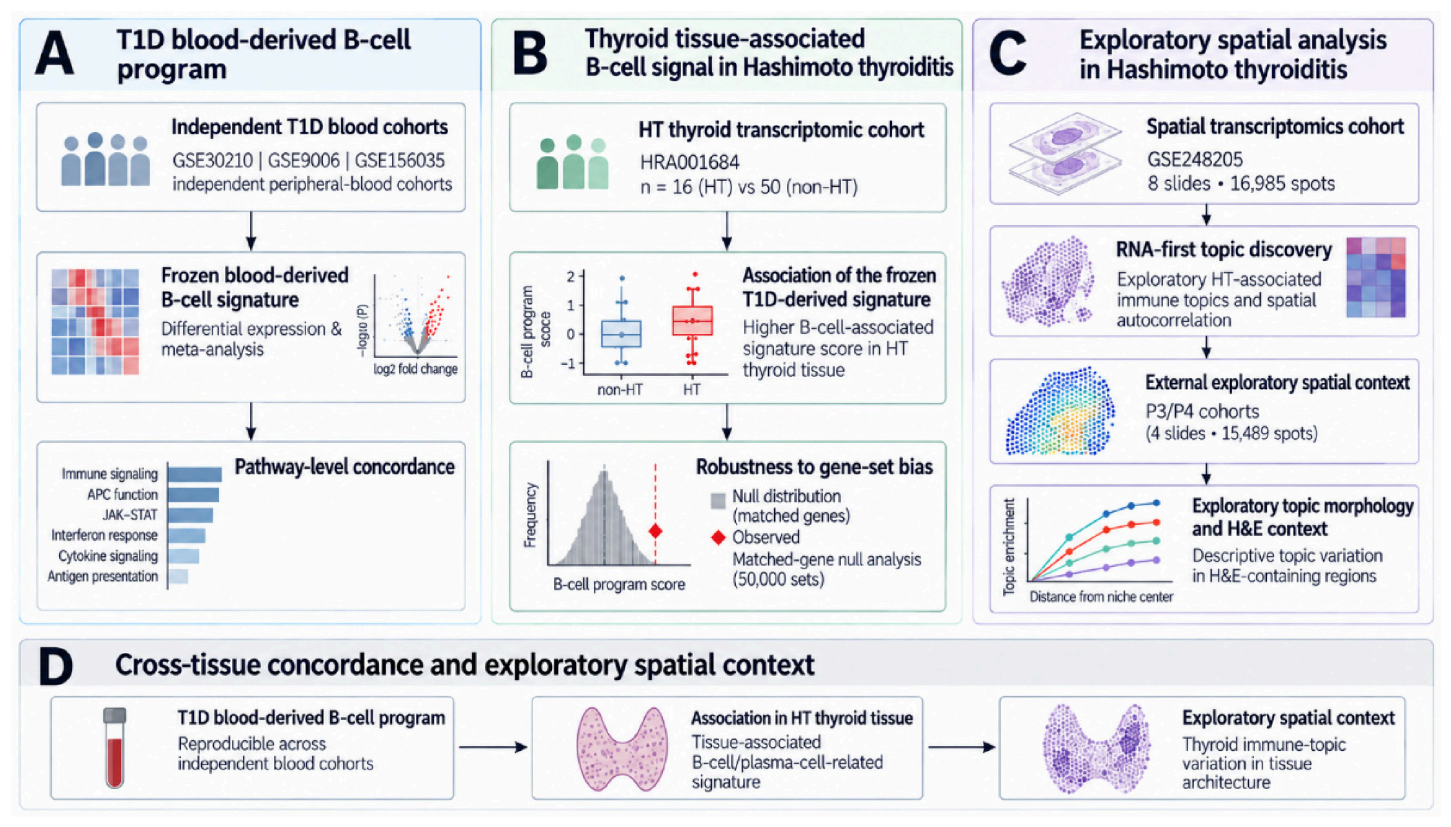
Study design and inferential boundaries of T1D blood-to-HT thyroid analysis. (**A**) Three T1D peripheral-blood cohorts (GSE30210, GSE9006, and GSE156035) contributed cohort-level B-cell effects to the meta-analysis and supplied the gene-level estimates used to freeze a separate 14-gene weighted signature. (**B**) The frozen score was associated with HT status in HRA001684 (16 HT, 50 non-HT; HC3-adjusted β = 2.6534, 95% CI 2.3679–2.9389). The historical 15-gene score is not presented as a separate validation result. The current fixed-score analysis uses one 66-sample HT cohort and does not constitute independent HT-cohort replication. (**C**) GSE192580 supplied descriptive thyroiditis immune-cell data (6512 cells; no healthy controls), GSE248205 supplied the five-slide HT/control spatial contrast (exact *P* = 0.10), and GSE230424 supplied external descriptive spatial-topic maps. RNA-first topics were inferred separately from the frozen score. (**D**) Transfer arrows denote application of fixed gene weights across datasets, not B-cell trafficking. HT blood activation, T1D islet expression, and preferential thyroid localization of the frozen signature were not established.

Figure 1 outlines the datasets and their distinct analytical roles. Three T1D peripheral-blood cohorts (GSE30210, GSE9006, and GSE156035) informed the retained pathway-level meta-analysis and the separately reported frozen 14-gene signature. HRA001684 provided the HT thyroid bulk cohort (16 HT; 50 non-HT) for the reported 14-gene scoring analysis; a historical 15-gene score is mentioned solely to explain study provenance. GSE192580 was used for descriptive thyroiditis single-cell profiling (6512 cells; three HT and three immune-checkpoint-inhibitor-associated thyroiditis libraries, without healthy controls). The GSE248205 discovery data comprised three HT, two control, and three Graves’ disease sections; only the five HT/control sections were included in disease-level spatial inference. GSE230424 supplied external descriptive maps from two HT-only sections (P3/P4) and two carcinoma-associated HT sections (P1/P2). RNA-first topic maps and exploratory H&E models described tissue patterns independently of the transferred signature.

### 2.2. Meta-Analysis of B-Cell-Associated Effects Across T1D Blood Cohorts

**Figure 2:**
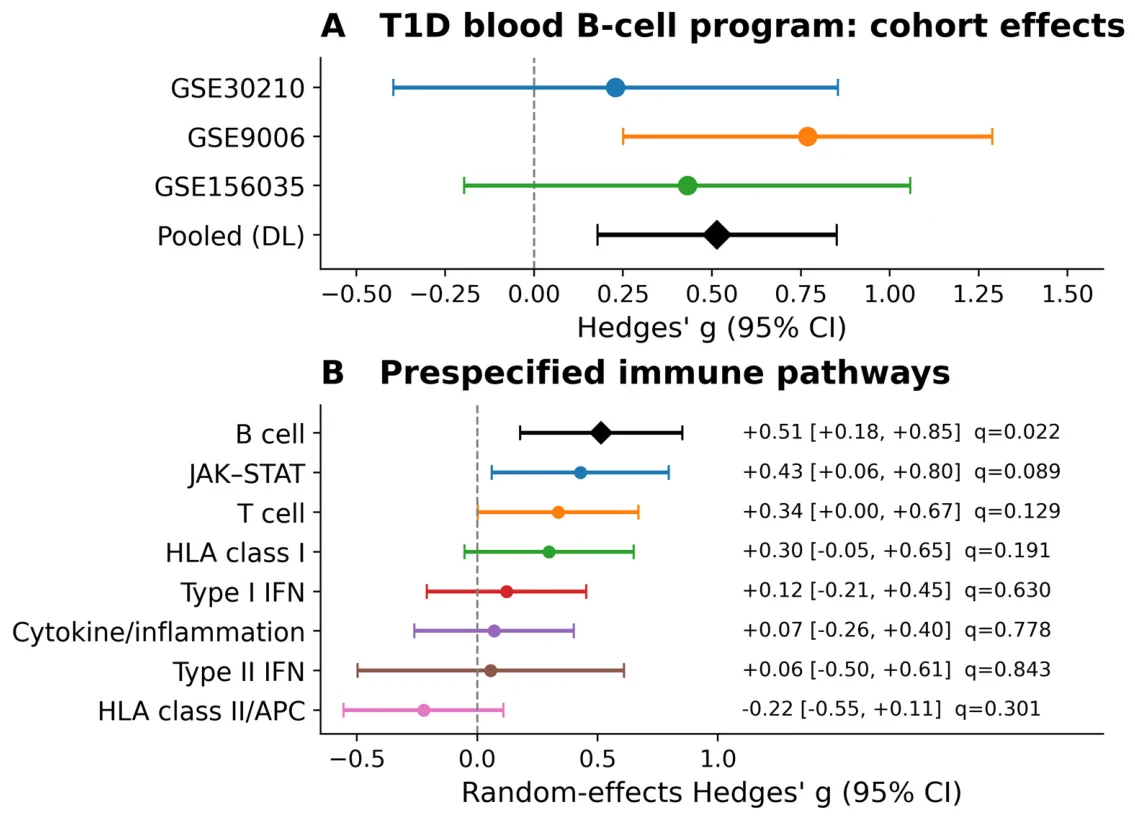
Meta-analysis of blood immune-program effects in T1D. (**A**) Cohort-specific Hedges’ g estimates and 95% confidence intervals for the B-cell program in GSE30210, GSE9006, and GSE156035. The DerSimonian–Laird pooled estimate was g = 0.515 (95% CI, 0.178–0.852; q = 0.0219; I^2^ = 0%). (**B**) Pooled effect estimates, 95% confidence intervals, and Benjamini–Hochberg (BH)-adjusted q values for eight immune pathways. These plots reproduce the meta-analysis from 24 retained cohort-level pathway-effect inputs, not the upstream generation of those effect estimates from individual-level expression matrices.

The B-cell-associated program showed positive effects across three T1D blood cohorts. Random-effects meta-analysis yielded Hedges’ g = 0.51 (95% CI 0.18–0.85; q = 0.022; I^2^ = 0%; Figure 2A), although one cohort-specific confidence interval included zero. B-cell and JAK–STAT pathways had the largest positive summary estimates; T-cell, HLA class I, type I interferon, and cytokine/inflammatory programs were also positive, whereas the HLA class II/APC estimate was weaker and negative (Figure 2B). Several pathway confidence intervals crossed zero. Figure 2A,B summarize 24 publicly released cohort-level pathway-effect inputs. An independent standard-library checker agrees with all eight retained pooled pathway results, but neither available script re-estimates the original effects from participant-level expression data.

### 2.3. Exploratory RNA-First Spatial Characterization of Immune-Associated Thyroid Topics

**Figure 3:**
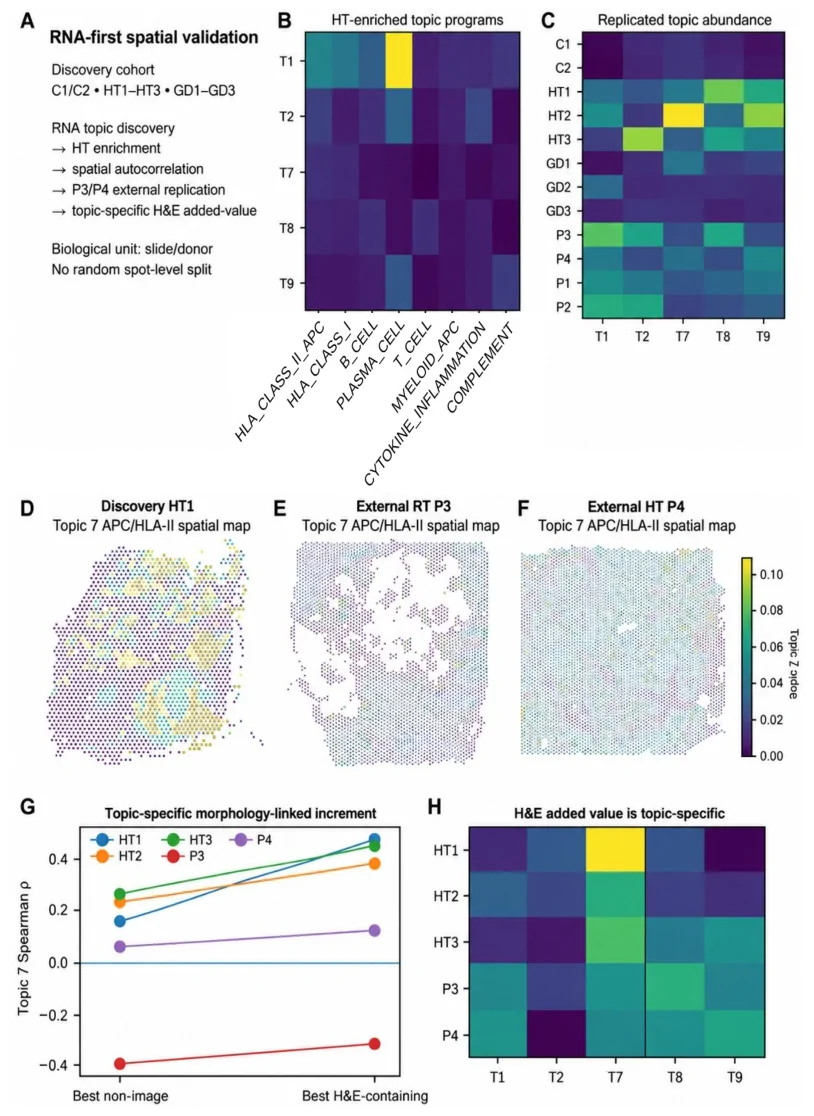
Exploratory thyroid RNA-first immune topics and their correspondence with histology. (**A**) RNA-first topic inference followed by descriptive disease comparisons, spatial autocorrelation assessment, external mapping, and exploratory H&E-associated analyses. The tissue section, rather than an individual spot, was the biological unit of analysis. (**B**) Transcriptional features of selected immune-associated topics T1, T2, T7, T8, and T9 across HLA class I, HLA class II/APC, B-cell, plasma-cell, T-cell, myeloid/APC, inflammation, and complement programs. (**C**) Relative topic abundances in eight GSE248205 discovery sections (controls C1/C2, *n* = 2; HT1–HT3, *n* = 3; Graves’ disease GD1–GD3, *n* = 3) and four GSE230424 external sections (HT only P3/P4, *n* = 2; papillary thyroid carcinoma with HT P1/P2, *n* = 2). (**D**–**F**) Descriptive maps of APC/HLA-II-associated Topic 7 scores in discovery HT1 (**D**) and external HT-only P3 (**E**) and P4 (**F**). P3/P4 were selected to avoid concurrent carcinoma; greater color intensity indicates a higher topic score, not localization of the frozen T1D signature. (**G**) Topic 7 Spearman correlation for the best non-image and H&E-containing models in HT1–HT3 and P3/P4; changes were heterogeneous. (**H**) Topic-specific H&E-associated changes for the five selected topics. These previously generated models and maps are exploratory, do not treat spots as independent biological replicates, and do not establish independent spatial replication of the frozen signature. Original topic/model selection and H&E fitting dependencies remain incompletely documented; the panels should not be interpreted as independently reproduced.

The previously generated RNA-first spatial topic outputs were reviewed descriptively, with tissue sections rather than individual spots as biological units (Figure 3A). The upstream topic-fitting, selection, and H&E-model histories were not independently reconstructed in the current public package. Transcriptomic topics were inferred before exploring disease contrasts, spatial autocorrelation, correspondence across datasets, and H&E-associated model performance. Five selected topics (T1, T2, T7, T8, and T9) showed overlapping but distinct B-cell, plasma-cell, HLA, antigen-presentation, inflammatory, and complement features (Figure 3B); T1 was particularly enriched for plasma-cell and HLA-II/APC annotations. Topic scores varied across discovery and external sections (Figure 3C). External GSE230424 comprised HT-only P3/P4 and papillary-thyroid-carcinoma-associated HT P1/P2. P3/P4 were chosen for illustrative maps because their noncarcinoma context more closely matched discovery HT1, not because they statistically replicated a signature effect. APC/HLA-II-associated Topic 7 scores varied within HT1, P3, and P4 (Figure 3D–F). The originally reported H&E-associated comparison showed numerically higher Topic 7 correlations in most evaluated sections, with heterogeneous changes and one negative external correlation (Figure 3G,H); these historical numbers were neither independently rerun nor evidence of predictive improvement. These are descriptive topic–morphology patterns, not evidence that the frozen T1D score occupies a particular anatomical region. Figure 3C displays eight discovery sections (two controls, three HT, and three Graves’ diseases) and four external sections (two HT only and two carcinoma-associated HT sections).

### 2.4. Frozen T1D Signature in HT Bulk Tissue and Descriptive Single-Cell Immune Context

**Figure 4:**
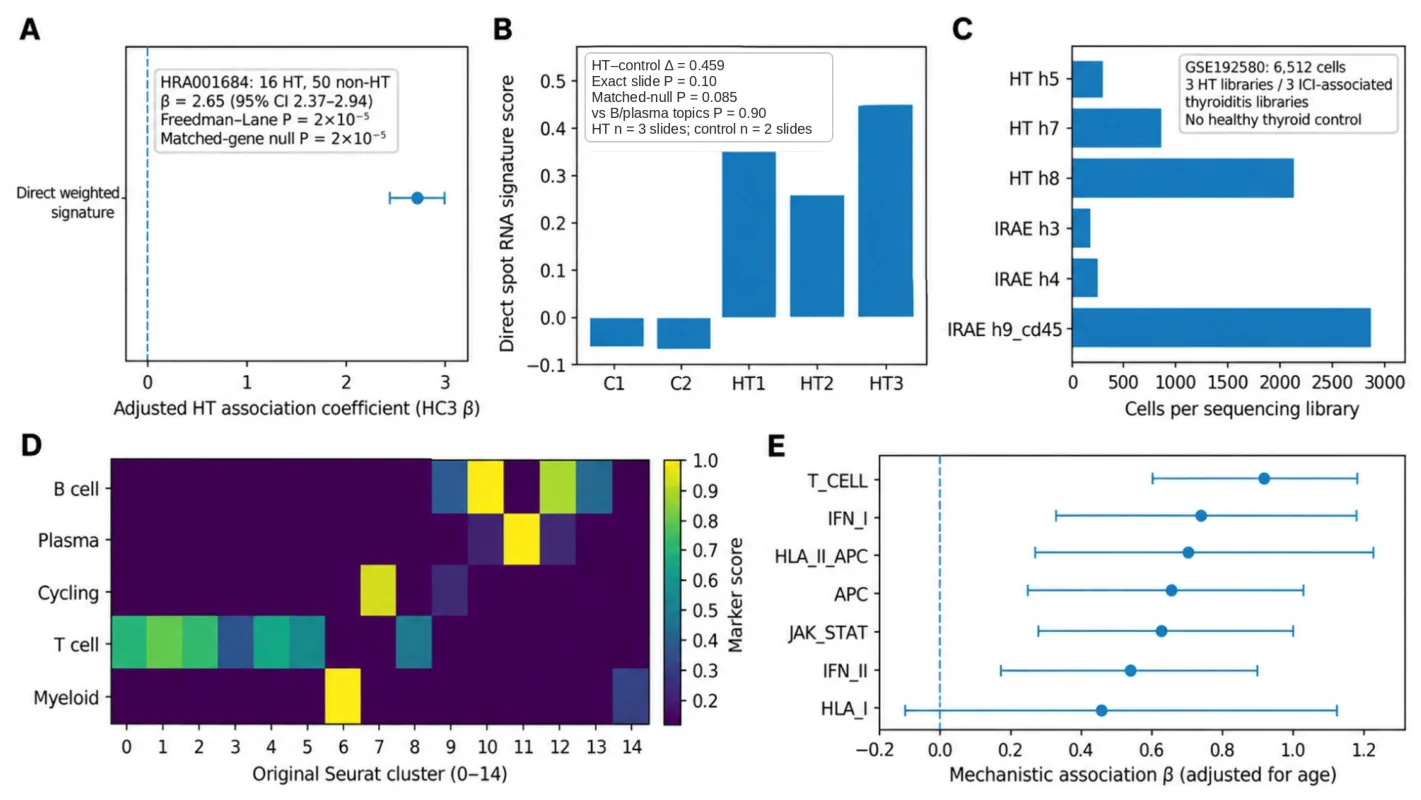
Frozen T1D-derived signature association in bulk thyroid tissue, exploratory spatial assessment, and descriptive single-cell immune context. (**A**) Association of the frozen 14-gene T1D blood-derived score with HT in HRA001684 (16 HT, 50 non-HT). The point and horizontal whiskers show the adjusted ordinary least-squares HT coefficient (β = 2.6534) and HC3 95% confidence interval (2.3679–2.9389); the dashed line marks β = 0. Freedman–Lane residual permutation and mean/variance-matched gene-null tests each used 50,000 draws and yielded empirical *P* = 2 × 10^−5^. These empirical *p* values are distinct from the regression confidence interval. (**B**) Frozen-signature scores in five GSE248205 spatial sections (HT1–HT3, *n* = 3; controls C1/C2, *n* = 2). The observed contrast (Δ = 0.459) was nonsignificant by exact slide-label permutation (*P* = 0.10) and matched-gene testing (*P* = 0.085); the previously reported correspondence with prespecified B/plasma-cell topics was also unsupported (*P* = 0.90). (**C**) GSE192580 processed library counts: 3292 cells from three HT libraries and 3220 cells from three immune-checkpoint-inhibitor-associated thyroiditis (ICI-thyroiditis) libraries. No healthy controls were included, and one ICI library (h9_cd45) pooled material from two individuals. (**D**) Descriptive B-cell, plasma-cell, proliferation, T-cell, and myeloid marker-gene expression across the 15 original Seurat clusters (0–14) of the same 6512 CD45-positive cells. Gene counts were normalized as log1p(10,000 × gene UMI/total cellular UMI); cluster markers were averaged across cells within each contributing library, then across libraries with equal weight. The heatmap contains no disease-effect estimates, confidence intervals, or tests, and individual cells were not treated as independent patients. (**E**) Complementary HT bulk immune-pathway associations in HRA001684, estimated using age-adjusted regression with HC3 covariance. Points show coefficients, and whiskers indicate 95% confidence intervals. Panels (**C**–**E**) provide reported descriptive context rather than independent validation or localization of the frozen score; source-to-output reconstruction is not demonstrated by the basic public code release. The exact slide-label *p* value is one-sided and is limited by only ten possible HT/control label allocations.

We derived a frozen weighted signature by inverse-variance pooling gene-level Hedges’ g estimates across GSE30210, GSE9006, and GSE156035, followed by L1 normalization of the pooled estimates. Fourteen of the 17 candidate genes were present in all three cohorts; their signed weights are reported in Supplementary Table S1. In HRA001684, the frozen score was associated with HT status (HC3-adjusted β = 2.6534, 95% CI 2.3679–2.9389; Figure 4A). Both the reported two-sided Freedman–Lane test and the reported 50,000-draw mean/variance-matched gene-null test yielded empirical *P* = 2 × 10^−5^; these results have not been independently rerun from the source matrices using the present basic repository. The earlier unweighted 15-gene program is retained only as methodological provenance, not as an additional association result or independent validation cohort. Spatial projection of the frozen score in GSE248205 gave a nonsignificant HT–control difference (Δ = 0.459; three HT and two control slides; exact slide-label *P* = 0.10; matched-gene null *P* = 0.085; Figure 4B). The previously reported correspondence with prespecified B/plasma-cell topics was also unsupported (*P* = 0.90). GSE192580 contained three HT and three immune-checkpoint-inhibitor-associated thyroiditis libraries (3292 and 3220 cells, respectively), without healthy controls; one ICI library pooled two individuals. Figure 4C reports processed library cell counts, and Figure 4D shows descriptive lineage-marker expression across the 15 original Seurat clusters. Figure 4E summarizes HT bulk pathway associations as additional biological context. None of these complementary panels establishes cellular or spatial localization of the frozen signature.

### 2.5. B-Cell Marker Adjustment and Signature-Weight Sensitivity Analyses

**Figure 5:**
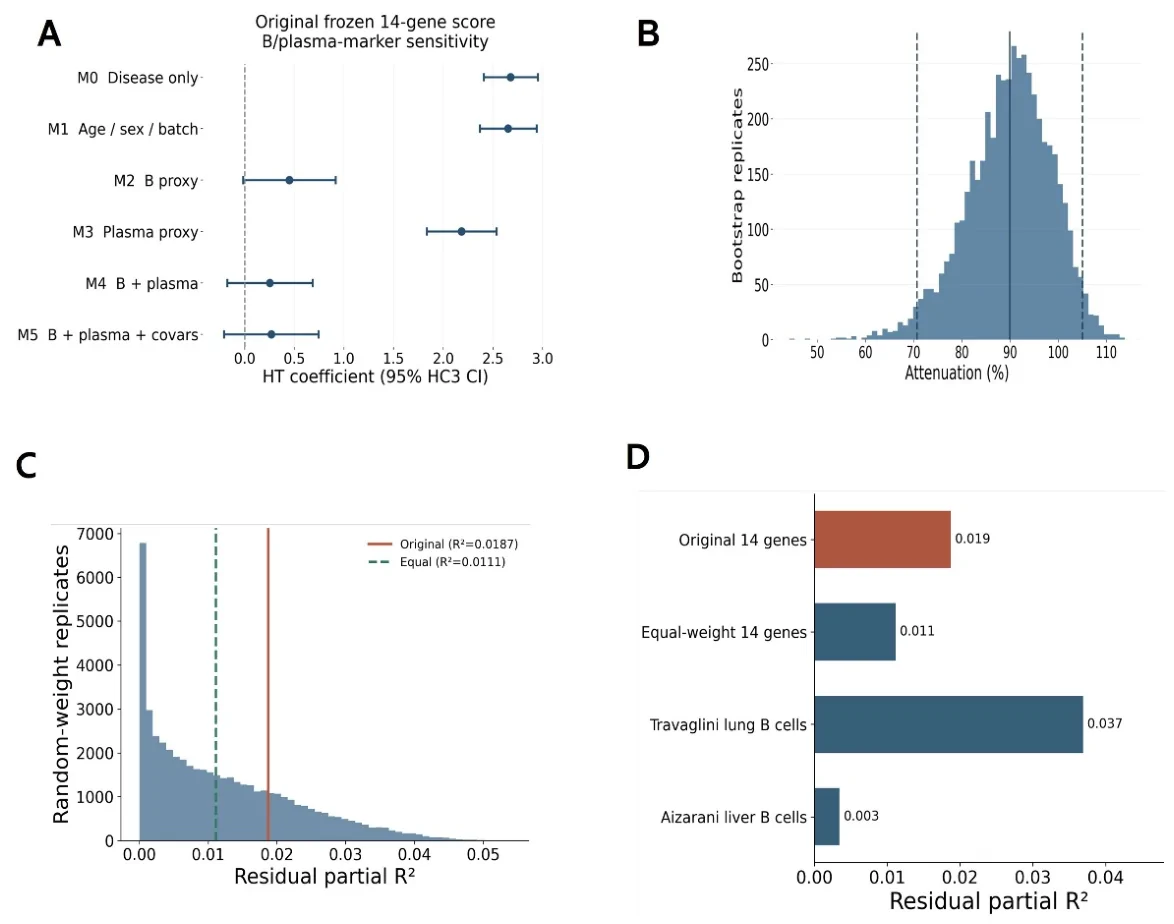
B-cell and plasma-cell marker-adjusted sensitivity analysis and signature-weight benchmarking of the frozen 14-gene T1D-derived signature in Hashimoto thyroiditis. (**A**) Forest plot showing HT-associated regression coefficients and heteroskedasticity-robust HC3 95% confidence intervals for the original frozen 14-gene signature across six models: disease status alone (M0), adjustment for age, sex, and library-preparation batch (M1), B-cell marker proxy (M2), plasma-cell marker proxy (M3), both marker proxies (M4), and both marker proxies plus clinical and technical covariates (M5). The vertical dashed line indicates a regression coefficient of zero. (**B**) Bootstrap distribution of the percentage attenuation in the HT-associated coefficient after combined B-cell and plasma-cell marker adjustment. The central estimate indicates approximately 90% attenuation, with variability across bootstrap replicates. (**C**) Distribution of residual partial R^2^ values obtained from random-weight benchmark replicates. Vertical lines indicate the observed residual partial R^2^ values for the original frozen-weight signature (0.0187; solid orange) and the equal-weight 14-gene signature (0.0111; dashed green). (**D**) Comparison of residual partial R^2^ values for the original frozen 14-gene signature (0.019), equal-weight 14-gene signature (0.011), Travaglini lung B-cell reference signature (0.037), and Aizarani liver B-cell reference signature (0.003). Residual partial R^2^ quantifies the incremental association with HT status after adjustment for the variables included in the corresponding benchmark models. Together, these analyses demonstrate substantial attenuation of the HT-associated signature coefficient following B-lineage marker adjustment and variability in residual association strength across alternative signature-weighting strategies and B-cell reference signatures. The sensitivity and weighting panels use the same HRA001684 cohort and do not constitute external patient-cohort validation. Bootstrap settings, random-weight generation, reference-score gene coverage, and relevant sample-level inputs remain necessary for independent reconstruction of the displayed numerical results. No statistically established advantage of the T1D-derived weights over equal weighting or alternative reference signatures is demonstrated.

To determine whether the association between the frozen 14-gene T1D-derived signature and Hashimoto thyroiditis (HT) was independent of B-lineage-associated transcriptional variation, we performed marker-proxy-adjusted sensitivity analyses. The original signature yielded an HT-associated regression coefficient after adjustment for age, sex, and library-preparation batch (β = 2.65, HC3 95% CI 2.37–2.94; Figure 5A). Adjustment for the B-cell marker proxy substantially reduced this coefficient (β = 0.45), whereas adjustment for the plasma-cell proxy alone produced a smaller reduction. Simultaneous adjustment for both marker proxies further attenuated the association, with the fully adjusted coefficient approaching zero and its confidence interval including zero. The reported bootstrap distribution indicated approximately 90% attenuation after joint B-cell/plasma-cell marker adjustment (Figure 5B); the bootstrap resampling specification and underlying sample-level inputs have not been independently verified in this revision. Because these scores are expression-based proxies, the attenuation cannot be interpreted as a measured reduction in B-cell abundance or as a causal mediation effect.

We next evaluated the sensitivity of the association to signature weighting. The original frozen 14-gene weights yielded a residual partial R^2^ of 0.0187, compared with 0.0111 for an equal-weight version of the same signature (Figure 5C). The reported random-weight benchmarks showed substantial variability in residual partial R^2^ values, but their sampling distribution and seed-level specifications require independent verification. Benchmarking against external B-cell reference signatures revealed a higher residual partial R^2^ for the Travaglini lung B-cell signature (0.037) and a lower value for the Aizarani liver B-cell signature (0.003), compared with 0.019 for the original frozen signature (Figure 5D). Taken as exploratory sensitivity results, these comparisons suggest that the reported HT association is strongly dependent on B-lineage marker variation and is sensitive to signature composition and weighting; they do not establish cell-specific causation. Accordingly, the transferred signature should be interpreted primarily as a tissue-associated B-cell transcriptional correlate rather than evidence of an HT-specific or cell-intrinsic disease program. The reference-signature benchmarks are exploratory comparisons of residual associations rather than head-to-head comparisons of tissue specificity or clinical predictive performance. The reported difference between the original frozen weights and equal weighting (partial R^2^, 0.0187 versus 0.0111) does not establish statistically significant superiority; the Travaglini reference score showed a larger residual partial R^2^ (0.037), and the reference-signature comparisons are not clinical predictive-performance tests.

## 3. Discussion

Across three T1D blood cohorts, the B-cell-associated program showed consistent summary-level effects, and a separately frozen 14-gene score was associated with HT status in bulk thyroid tissue. This reported fixed-score transfer is the principal biological observation, but it requires independent reconstruction of the frozen weights, expression standardization, and sample-level HT scoring for full computational reproducibility. The single-cell and RNA-first spatial analyses characterize related immune features, but neither directly tests the cellular origin or spatial localization of that transferred score. The findings therefore support partial transcriptional concordance across the sampled compartments, not a demonstrated common circulating B-cell state or causal link between the diseases. Marker-proxy adjustment substantially attenuated the reported HT association, and the available benchmark results do not demonstrate superior performance for the T1D-derived weights.

The pooled T1D B-cell effect was positive, with no detectable heterogeneity across the three retained cohort estimates. This consistency is relevant because platform, sampling, and disease-stage differences can influence individual transcriptomic analyses. B cells have established functions in T1D antigen presentation and adaptive immunity [5,6,19]. Rituximab-mediated CD20-positive B-cell depletion transiently preserved endogenous β-cell function in recent-onset T1D, although it did not produce sustained disease modification [20]. Variation in CD20-positive infiltration across pancreatic pathological subtypes further cautions against interpreting the blood-associated program as uniform across all patients [15,16].

The B-cell-associated signal occurred alongside JAK–STAT, T-cell, HLA class I, and interferon-related pathway associations. Interferon–JAK–STAT signaling can increase pancreatic β-cell HLA class I expression [4], while B cells may support autoreactive T-cell responses through antigen presentation [5,6]. The program is therefore best viewed within an interacting immune network rather than as an isolated B-cell-specific mechanism.

The fixed weighted signature was associated with HT status in bulk thyroid tissue. The matched-gene analysis evaluates this association relative to the specified competitive null and does not constitute independent patient-cohort replication. HT is characterized by local B-cell infiltration, antigen presentation, and germinal-center-like aggregates [7,8,10,21,22]. Such processes, including differences in immune-cell abundance, could contribute to the bulk thyroid association without systemic activation of the same score in HT. In the reported marker-proxy models (Figure 5A–B), the HT coefficient was attenuated by approximately 90% after joint B-cell/plasma-cell marker adjustment, and the fully adjusted confidence interval included zero. This finding indicates sensitivity to cellular composition or related transcriptional variation but does not distinguish cell abundance from activation and does not establish causal mediation.

Shared susceptibility factors, including HLA, CTLA4, and PTPN22, do not imply identical disease mechanisms [3,23]. T1D and HT differ in target cells and local effector responses, as reflected in comparative immune-cell studies [12]. Without matched blood–thyroid samples or B-cell clonotypes, the thyroid score cannot distinguish circulating-cell recruitment, local expansion, or changes in cell composition.

The frozen score was derived from genes shared across three T1D blood cohorts, with fixed signed weights transferred without optimization against HT labels (Supplementary Table S1). The reported HT association (β = 2.65, 95% CI 2.37–2.94; matched-gene *P* = 2 × 10^−5^) represents a single-cohort cross-dataset application, not an independently reproduced or externally replicated result. Alternative scores evaluated in the same cohort do not provide independent replication. Robustness to exclusion of individual genes or T1D discovery cohorts was not assessed by leave-one-gene-out or leave-one-cohort-out analyses.

The GSE192580 reference was suitable for describing immune-lineage markers, not for an HT-versus-healthy-control test. Its three HT and three ICI-associated thyroiditis libraries included one pooled ICI specimen. B-cell markers were evident in Seurat clusters 10 and 12, plasma-cell markers in cluster 11, and other clusters showed proliferative, T-cell, or myeloid features. These descriptive panels do not establish an HT-versus-healthy disease effect or independently confirm the transferred signature.

RNA-first spatial topics varied among thyroid sections and included an APC/HLA-II-associated profile (Topic 7). In GSE230424, HT-only P3/P4 were selected for representative maps instead of carcinoma-associated HT P1/P2 to improve pathological comparability. Their visually heterogeneous scores did not statistically replicate a frozen-signature distribution. The patterns are compatible with known intrathyroidal lymphoid aggregates, stromal responses, and antigen-presentation networks [10,21,22], as well as previously reported spatial heterogeneity [11]. H&E-derived information contributed unevenly across topics and sections, underscoring the distinction between morphology and transcriptomic scores.

The direct five-slide spatial test of the frozen signature was nonsignificant (Δ = 0.459; exact slide-label *P* = 0.10; matched-gene *P* = 0.085), and the originally reported B/plasma-topic correspondence was unsupported (*P* = 0.90). APC/HLA-II-associated Topic 7 was inferred independently and cannot be treated as a map of the transferred score. The exact test had only ten possible HT-label allocations, limiting its resolution, but this does not justify a positive localization claim. Additional independent sections and prespecified spatial tests are needed.

The thyroid association may reflect convergent inflammatory pathways, local B-cell abundance, or cell-intrinsic activation. In T1D, interferon signaling, HLA class I hyperexpression, and cytotoxic injury are prominent [4]; in HT, infiltrating B cells, antigen-presenting cells, stromal activation, and thyrocyte–immune interactions contribute to inflammation [7,10,21]. The present bulk and descriptive spatial data cannot distinguish among these explanations.

This study is an integrative computational and translational analysis, not a new bioinformatics algorithm or a clinical diagnostic validation. The proposed reusable element is the fixed 14-gene definition and signed weights reported in Supplementary Table S1, together with a cross-cohort evaluation strategy; however, the present public release does not provide a verified end-to-end source-to-score implementation. External application should retain the original gene definitions and weight directions only after the normalization rules, missing-gene handling, and scoring workflow have been independently verified; effects should be estimated at the participant or specimen level. The earlier 15-gene score is distinct from the fixed 14-gene signature.

The thyroid bulk association motivates tests of whether B-cell abundance, activation, or antigen presentation contributes to the measured score. Prospective studies could collect contemporaneous blood and thyroid data from patients with T1D, HT, or both, together with thyroid autoantibodies, thyroid function, glycemic measures, clinical phenotypes, and longitudinal outcomes. The fixed score could then be evaluated for incremental value over established variables. B-cell receptor sequencing and functional perturbation experiments may help distinguish shared immune states from local infiltration. These are research directions: diagnostic accuracy, prognosis, treatment-response prediction, and therapeutic utility were not assessed.

Important biological gaps remain. We did not analyze the frozen signature in HT peripheral blood, matched blood–thyroid specimens, or T1D pancreatic-islet transcriptomes. Published HT blood profiles [9], pancreatic pathology reports [15,16], and islet atlases [17,18] offer potential sources of evidence but were not subjected to the present scoring pipeline. Paired sampling, suitable controls, cell-type-aware modeling, and B-cell receptor profiling would help test compartment concordance and cellular origin.

An islet-specific follow-up should document B-lineage cell and donor counts, detection of all 14 genes, and application of the unchanged signed weights. Where feasible, donor-level or pseudobulk T1D–control comparisons should be paired with independent spatial or histological evaluation of insulitic lesions. Neither islet expression nor blood–islet localization can be inferred from the current data.

Several methodological constraints limit interpretation. The HT bulk analyses use the same 66 HRA001684 specimens, and marker-proxy adjustment does not distinguish cell abundance from activation or establish causal mediation. GSE192580 lacks healthy controls and includes a pooled ICI library. Spatial inference used only three HT and two control sections; the external illustrative maps represent the HT-only subset of a collection that also contains carcinoma-associated HT. Topic selection, H&E comparisons, and the Figure 5C–D benchmarks are exploratory. Available documentation does not fully specify the original pathway-level visit estimator, exact standardization helper, spatial topic/model-selection history, bootstrap settings, random-weight distribution, and reference-score coverage rules. These unresolved input- and analysis-lineage gaps prevent a claim of independent end-to-end reproduction of Figure 3, Figure 4 and Figure 5 and limit the confidence that can be placed in the corresponding numerical comparisons. Neither the T1D-derived weights nor an alternative reference signature has demonstrated statistically established superiority in these comparisons. The public reproducibility scope is specified in Section 4.12.

## 4. Materials and Methods

### 4.1. Study Design and Public Transcriptomic Datasets

This secondary analysis integrated public human transcriptomic datasets to evaluate systemic T1D B-cell-associated transcription, a cross-tissue association in a separate HT bulk cohort, descriptive immune-cell features, and exploratory variation in thyroid spatial immune-topic scores. The systemic-to-thyroid evaluation concerns population-level cross-dataset associations, not paired blood–thyroid observations from the same individuals. The two HT bulk scoring procedures use the same HRA001684 individuals, and therefore do not constitute two independent validation cohorts. The 15-gene early exploratory B-cell definition, 17-gene extended candidate list, and 14-gene frozen signature are explicitly distinguished, and spatial topic distributions are not equated with localization of the frozen systemic score. HT peripheral blood, paired blood–thyroid samples, and T1D pancreatic-islet expression data were not analyzed.

Systemic analyses used GSE30210, GSE9006, and GSE156035 as separate expression cohorts. The retained pathway summary reports case/control entries of 18/22, 42/24, and 20/20, respectively. HRA001684 contains 16 HT and 50 non-HT thyroid specimens. GSE192580 comprises six thyroiditis libraries (three HT and three ICI-associated thyroiditis), without a healthy-control group. GSE248205 comprises two control, three HT, and three Graves’ disease sections, although the direct HT–control inference used only five slides. GSE230424 comprises HT-only P3/P4 and papillary-thyroid-carcinoma-associated HT P1/P2 sections. The GSE30210 pathway-table counts describe analysis records; participant-level and visit-level counts are not interchangeable. Participants, libraries, and slides were treated as distinct biological units according to the analysis; single cells or spatial spots were not considered independent disease-level replicates. Dataset-specific processing and provenance are detailed in Supplementary Methods S1–S3.

### 4.2. Gene Harmonization and Versioned B-Cell Programs

Gene identifiers were harmonized to human gene symbols. In the documented frozen-signature procedure, duplicate mapped gene symbols were collapsed by the median, followed by gene-wise robust standardization. The exact shared-helper implementation, input-to-gene mapping decisions, and upstream platform-specific normalization are not fully specified in the available public documentation. Therefore, the reported 14-gene signature cannot yet be regenerated independently from the original cohort expression matrices.

The initial exploratory B-cell module comprised BANK1, BLK, CD19, CD22, CD27, CD37, CD40, CD79A, CD79B, CD83, IGHM, IGKC, JCHAIN, MS4A1, and MZB1 (15 genes). TNFRSF13B and TNFRSF17 were subsequently added to form a 17-gene systemic candidate set. The frozen weighted signature retained only 14 genes measurable in all three systemic T1D cohort matrices, excluding IGHM, IGKC, and JCHAIN. These stage-specific sets are not interchangeable; the early 15-gene module is not the same quantity as the 14-gene weighted score. The complete version crosswalk and all 14 signed coefficients are provided in Supplementary Table S1 and Supplementary Methods S2. The provenance audit supports the term “exploratory” for the initial B-cell program rather than treating it as prospectively prespecified across all versions.

### 4.3. Cross-Cohort Analysis of Systemic T1D Immune Programs

For the documented frozen-weight workflow, repeated GSE30210 observations were reduced to the earliest eligible visit per participant (or the first eligible record if visit order was unavailable); other repeated subject identifiers in this procedure were summarized by the within-person median. For each retained gene, T1D–control Hedges’ g estimates and sampling variances were pooled across three cohorts by fixed-effect inverse-variance weighting. The separately retained eight-pathway, three-cohort summary table was synthesized by DerSimonian–Laird random-effects meta-analysis, followed by Benjamini–Hochberg correction.

The summary-table reconstruction yielded a B-cell pooled Hedges’ g of 0.514716 (95% CI, 0.177885–0.851547; *P* = 0.002744; q = 0.021948; I^2^ = 0%). The earliest-visit rule belongs to the frozen-signature derivation, whereas a separate composition-adjusted sensitivity script used subject-wise medians. The executable summary-level workflow begins with the 24 retained cohort–pathway effect estimates; it does not reconstruct participant inclusion, visit selection, preprocessing, or effect estimation from original expression matrices (Supplementary Methods S3 and S4).

### 4.4. Cross-Tissue Evaluation in Hashimoto Thyroid Tissue

The historical unweighted 15-gene program and the fixed 14-gene weighted signature are distinct scoring definitions. Only the latter is retained as the quantitative cross-tissue association in the revised manuscript (HRA001684: 16 HT and 50 non-HT specimens). The present cross-tissue interpretation focuses on the fixed 14-gene score; the earlier 15-gene result is not treated as additional confirmatory evidence or independent cohort replication. The scoring and regression procedures for the 14-gene analysis are described in Section 4.5.

### 4.5. Frozen T1D-Derived Signature and Cross-Tissue Transfer

The frozen signature was constructed from the 17-gene systemic candidate panel by retaining 14 genes shared across GSE30210, GSE9006, and GSE156035. The cohort-specific Hedges’ g estimates for each gene were pooled with inverse sampling-variance weights, then normalized as w_g = m_g/Σ_h |m_h|, where m_g denotes the pooled effect for gene g. The frozen-signature score was calculated as S_i = Σ_g w_g Z_gi, with Z_gi representing source-standardized expression for gene g in sample i. All 14 observed weights were positive; their exact values and the 15 → 17 → 14 provenance are reported in Supplementary Table S1. Exact robust-standardization and missing-gene rules remain incompletely specified in the released materials (Supplementary Methods S2 and S5); providing signed weights alone is insufficient to regenerate the published sample scores and inferential statistics.

The HRA001684 analysis collapsed duplicate gene symbols by their median, applied log2(expression + 1), and then invoked gene-wise robust standardization. The reported covariate-adjusted OLS model included HT status, age, sex, and library-preparation batch, consistent with the model reported in Section 2.5 and Figure 5A. HC3 inference gave an adjusted HT coefficient of β = 2.653394 (95% CI, 2.367850–2.938938). The originally reported 50,000 Freedman–Lane permutations yielded *P* = 1.99996 × 10^−5^; independent recomputation requires the covariate matrix, reduced-model fitted values/residuals, randomization implementation, and source-processed signature scores.

The matched-gene competitive null sampled replacement genes by joint deciles of mean expression and variance; matching pools were expanded to neighboring deciles where fewer than five non-signature candidates were present. Each signature-gene position was sampled independently, so a replacement gene could recur in a null gene set. Both null procedures used 50,000 draws and a two-sided empirical tail calculation of (1 + #{|T_null| ≥ |T_obs|})/50,001, yielding a weighted matched-gene *P* = 1.99996 × 10^−5^. These probabilities quantify distinct permutation and competitive-null tests and should not be conflated with the HC3 model-based confidence interval (Supplementary Methods S5 and S6).

For the exploratory B-cell/plasma-cell transcriptional proxy sensitivity analysis in HRA001684, the B-cell proxy included 12 detected marker genes (PAX5, CD72, CD180, FCRL1, FCRL2, FCRL5, FCER2, SPIB, TCL1A, BLNK, HVCN1, and TNFRSF13C), and the plasma-cell proxy included nine detected marker genes (PRDM1, XBP1, SDC1, DERL3, FKBP11, SEC11C, IRF4, JCHAIN, and TXNDC5). Neither marker panel overlapped with the frozen 14-gene T1D-derived signature. IGHA1 and IGHG1 were excluded because they were unavailable in the analyzed expression matrix. These expression-derived proxies do not directly quantify cell fractions or cell-intrinsic transcriptional activity. The six models displayed in Figure 5A comprise an HT-only model (M0), a model adjusted for clinical and technical covariates (M1), B-cell proxy adjustment (M2), plasma-cell proxy adjustment (M3), joint proxy adjustment (M4), and joint proxy plus clinical/technical covariate adjustment (M5). These comparisons were conducted within the same 66-sample thyroid cohort, and the proxies represent surrogate expression scores rather than measured cellular fractions.

### 4.6. RNA-First Spatial Topic Discovery and Biological Annotation

An exploratory RNA-first topic representation was obtained from eight GSE248205 spatial sections (C1, C2, HT1–HT3, and GD1–GD3).

The topic representation comprised 12 components; five immune-associated components (T1, T2, T7, T8, and T9) were selected for descriptive characterization. Topic rank and component selection were exploratory rather than prospectively prespecified; the original fitting configuration, initialization, seed, and selection record are not currently sufficient to verify the displayed maps by an independent rerun. Biological labels, including APC/HLA-II, describe topic-associated expression and do not establish localization of the fixed 14-gene signature. HT–control comparisons used slides, not spots, as independent units (Supplementary Methods S7).

### 4.7. External Spatial Topic Mapping

The external GSE230424 collection comprised HT-only sections P3/P4 and papillary-thyroid-carcinoma-associated HT sections P1/P2. P3/P4 were selected for illustrative maps because they most closely matched the HT-only disease context; all four sections were considered in the descriptive topic-profile comparison. Topic correspondence was assessed over shared mapped genes using cosine similarity of topic loading vectors, without assuming numerical topic IDs are comparable across datasets.

The external Topic 7 patterns represent descriptive APC/HLA-II-associated spatial context, not independent replication of frozen T1D B-cell signature localization. Original source-to-topic objects and cross-dataset topic-match uncertainty, alternative ranks, and refit stability were not fully characterized in the released evidence; no quantitative matching-confidence claim or disease-effect replication is inferred from these images (Supplementary Methods S8).

### 4.8. Exploratory H&E-Associated Added-Value Analysis

Previously reported H&E analyses compared non-image and H&E-containing prediction models for RNA-first topics under a stated leave-one-slide-out scheme; the original feature construction, model selection, fitted objects, and held-out predictions have not been independently verified. For each topic and held-out slide, added value was summarized as Δρ = ρ(H&E-containing model) − ρ(non-image model), where ρ denotes Spearman’s rank correlation.

H&E-associated differences were summarized descriptively across topics and held-out sections. These comparisons do not establish statistically significant improvements, independently validated generalization gains, or preferential spatial localization of the transferred B-cell signature (Supplementary Methods S9).

### 4.9. Statistical Analysis and Reproducibility

Study-level pathway effects were pooled by DerSimonian–Laird random-effects synthesis, with Benjamini–Hochberg correction across the eight reported programs. The HRA001684 weighted-score regression used HC3 robust covariance. The manuscript reports 50,000 Freedman–Lane residual permutations and 50,000 competitive matched-gene draws as distinct historical analyses; those source-to-result workflows have not been independently rerun from the public GitHub inputs. Exact HT-versus-control slide-label inference used three HT and two control sections, yielding ten possible assignments of the three HT labels.

The retained direct spatial result was an HT-minus-control slide-mean contrast of 0.458565, with exact one-sided label-permutation *P* = 0.10 and a matched-gene null *P* = 0.08548. The historical B/plasma-topic comparison (*P* = 0.90) also does not support preferential spatial correspondence. Spatial empirical *p* values, bulk-regression HC3 intervals, and descriptive H&E correlation differences answer different questions and must not be pooled into a single confirmatory claim. The analysis-specific reproducibility scope is described in Section 4.12 (Supplementary Methods S1–S10).

### 4.10. Marker-Proxy and Signature-Weight Sensitivity Analyses

Figure 5A summarizes the reported ordinary least-squares HT coefficients with HC3 robust confidence intervals across models M0–M5 (defined in Section 4.5). The reported percentage attenuation compares the HT coefficients before and after B-lineage marker-proxy adjustment; bootstrap resampling was reported to assess its variability (Figure 5B), although the resampling unit, iteration count, seed, and complete covariate-by-sample table must be verified to rerun this panel. Neither the regression coefficient attenuation nor the marker-derived proxy score constitutes a direct estimate of the infiltrating B-cell proportion or a causal mediation fraction.

Figure 5C,D compare the reported residual partial R^2^ for the original 14-gene weights, an equal-weight 14-gene score, random-weight benchmarks, and Travaglini lung and Aizarani liver B-cell reference signatures. Residual partial R^2^ describes a conditional incremental association after the covariates used in the corresponding benchmark model, rather than overall model fit. The comparisons are exploratory and do not establish superiority of the T1D-derived weights or constitute external patient-cohort validation. The original weight-draw distribution, random seeds, reference-signature gene lists/coverage, normalization, and model matrices must be supplied or verified before the numerical benchmarks can be independently reconstructed; their limitations are also described in the Discussion.

### 4.11. Scope of Sensitivity Analyses

The sensitivity analyses comprise marker-proxy adjustment and comparisons with alternative weighting schemes and reference signatures. Leave-one-gene-out and leave-one-cohort-out analyses of the transferred fixed signature are not reported; robustness to exclusion of individual genes or discovery cohorts is therefore not established.

### 4.12. Code Availability

The public manuscript-specific repository is https://github.com/sanghunlee-inha/T1D-HT-B-cell-program-reproducibility (accessed on 27 October 2026). The publicly executable scripts perform DerSimonian–Laird random-effects pooling and Benjamini–Hochberg correction using 24 retained cohort–pathway effect estimates, with an independent eight-pathway numerical consistency check (scripts/98_validate_summary_level.py); they do not reconstruct the underlying effects from source-expression matrices. A separately published Zenodo software archive (DOI: 10.5281/zenodo.23164148, version v1) contains additional analysis scripts for HT regression, matched-gene nulls, spatial permutation, topic mapping, single-cell analysis, and H&E-related analyses. The presence of these historical scripts does not establish complete end-to-end reproduction. The updated R3 reproducibility materials have been publicly archived on Zenodo as version v2 (DOI: 10.5281/zenodo.23715560; https://doi.org/10.5281/zenodo.23715560). This published version preserves the earlier v1 software ZIP together with the revised GitHub repository snapshot, including the figure-level audit and code/data availability documentation; archival publication does not establish independent end-to-end reproduction of Figure 3, Figure 4 and Figure 5. Reconstruction of the other retained quantitative results requires additional verified source-processed matrices, signed weights and standardization code, matching/null inputs, model-selection histories, resampling configurations, and/or model objects. The analysis-by-analysis evidence status and unmet dependencies are documented in README.md, docs/FIGURE_REPRODUCIBILITY_MATRIX.md, docs/REPRODUCIBILITY_STATUS.md, and docs/R3_MANUSCRIPT_ALIGNMENT.md (GitHub commit 721be5891874029186cff92bb143d906f28fbf11). The exact signed 14-gene coefficients are described in Supplementary Table S1; the public repository does not by itself establish their verified availability. Source data remain subject to the originating repositories’ access conditions.

## 5. Conclusions

A fixed 14-gene signature derived from T1D blood cohorts was associated with HT bulk thyroid status. Thyroiditis single-cell analyses described immune-lineage markers without healthy controls, and separately inferred spatial topics showed regional heterogeneity. The five-slide frozen-score contrast was not significant (exact *P* = 0.10; matched-gene *P* = 0.085), and preferential correspondence to B/plasma-cell topics was unsupported (*P* = 0.90). These reported observations are consistent with partial blood-to-thyroid transcriptional concordance but do not establish systemic HT B-cell activation, localization of the transferred score, a shared causal mechanism, or independent end-to-end replication of the HT association. The frozen score was not tested in HT blood or T1D islets. In the marker-proxy sensitivity analysis, joint adjustment attenuated the HT coefficient by approximately 90%, and the confidence interval for the fully adjusted coefficient included zero. Comparisons with equal weighting and external B-cell reference signatures did not demonstrate superiority of the T1D-derived signature weights. For biomedical researchers, the fixed score offers a hypothesis for external-cohort evaluation once its signed weights, gene normalization, and complete executable scoring workflow have been verified and released. Clinically oriented studies may evaluate it prospectively in paired or longitudinal blood–thyroid cohorts alongside standard diabetes and thyroid measurements. Independent validation and evidence of incremental clinical value are required before considering diagnosis, risk stratification, prognosis, or treatment selection. The current executable public workflow supports only the retained-effect meta-analysis and not end-to-end replication of the reported HT, spatial, or marker/weight findings.

## Data Availability

Source datasets are identified by GEO accessions GSE30210, GSE9006, GSE156035, GSE192580, GSE248205, and GSE230424, and GSA-Human accession HRA001684. Access is subject to the conditions of the respective repositories. The fixed 14-gene weight table is provided in Supplementary Table S1. The GitHub repository https://github.com/sanghunlee-inha/T1D-HT-B-cell-program-reproducibility supplies code and retained inputs for an executable summary-level T1D meta-analysis. A separate historical software archive is publicly available on Zenodo (DOI: 10.5281/zenodo.23164148, v1). The third-revision reproducibility package has been published and archived on Zenodo as version v2 (DOI: 10.5281/zenodo.23715560; https://doi.org/10.5281/zenodo.23715560), containing the revised GitHub snapshot and the earlier v1 software ZIP. The deposited scripts verify summary-level consistency for the eight T1D pathway estimates, but the archive does not establish complete source-to-result reproduction of the remaining analyses. Other reported analyses, including Figure 3, Figure 4 and Figure 5, are not established as independently reproducible from the deposited materials. Figure-specific missing inputs and dependencies are identified in Section 4.12 and the GitHub docs/FIGURE_REPRODUCIBILITY_MATRIX.md audit.

## Supplementary Materials

The following supporting information can be downloaded at: https://www.mdpi.com/article/10.3390/ijms27219698/s1.
