# Supplementary material for: Cross-Tissue Concordance of a T1D Blood-Derived B-Cell Signature with Hashimoto Thyroiditis Tissue: Exploratory Spatial Immune-Topic Analysis
Source: Int J Mol Sci. 2026 Oct 29;27(21):9698. doi: 10.3390/ijms27219698 (PMC13763269; doi:10.3390/ijms27219698)
Supplement: Supplementary file 1 [file ijms-27-09698-s001.zip › ijms-4640479-supplementary.pdf]

Supplementary Methods

S1. Dataset provenance, scope and analysis units

Systemic transcriptomic cohorts were GSE30210, GSE9006 and GSE156035. The retained pathway-level input table comprised eight immune programs across the three cohorts (24 rows), with case/control entries of 18/22, 42/24 and 20/20. These counts refer to historical analytical summaries and do not by themselves verify the number of independent participants, visits or disease contrasts in each original expression matrix. The HT bulk dataset HRA001684 contained 66 samples (16 HT, 50 non-HT). The processed GSE192580 single-cell analysis comprised three HT and three immune-checkpoint-inhibitor-associated thyroiditis libraries, with no healthy control; one of the latter libraries was pooled. Spatial dataset GSE248205 contained two control, three HT and three Graves' disease sections, while the direct HT–control test used three HT and two control slides. External spatial GSE230424 contained two HT-only sections (P3/P4) and two papillary-thyroid-carcinoma-associated HT sections (P1/P2). The participant, bulk specimen, cell-sequencing library and spatial tissue slide represent distinct observational units and are not interchangeable.

S2. Versioned B-cell gene programs and frozen-weight derivation

The initial exploratory 15-gene B_CELL list was BANK1, BLK, CD19, CD22, CD27, CD37, CD40, CD79A, CD79B, CD83, IGHM, IGKC, JCHAIN, MS4A1 and MZB1. TNFRSF13B and TNFRSF17 were added in a later systemic 17-gene candidate set. For the frozen weighted analysis, the intersection of genes present in all three T1D cohort matrices retained 14 genes; IGHM, IGKC and JCHAIN were absent from this intersection. These stages describe different analytic definitions and should not all be called a single prespecified 15-gene signature.

For retained gene g and T1D cohort k, the recovered weight-generation table records the Hedges' g contrast g_gk and its sampling variance v_gk. The fixed-effect gene-level pooled value is m_g = [Σ_k(g_gk/v_gk)]/[Σ_k(1/v_gk)]. The frozen signed coefficient is w_g = m_g / Σ_(h∈G14)|m_h|. All 14 observed coefficients are positive and their sum is approximately one. The retained A1 gene-by-cohort effects (42 rows) independently regenerate the A2 frozen weight table to machine precision, but this calculation does not independently reconstruct cohort-specific expression inputs.

Frozen 14-gene coefficients (full precision; identical to Supplementary Table S1)

Gene symbol	Frozen signed weight
**BANK1**	0.0244263551105605
**BLK**	0.0449279004980594

**CD19**	0.0867655576165349
**CD22**	0.0531703083447799
**CD27**	0.1178245546437827
**CD37**	0.0371399451794375
**CD40**	0.0387641480241145
**CD79A**	0.0640090037624852
**CD79B**	0.0678894825680535
**CD83**	0.0139391042717626
**MS4A1**	0.0881797191876886
**MZB1**	0.1307370543607531
**TNFRSF13B**	0.0738900375996495
**TNFRSF17**	0.1583368288323373

For target specimen i, the conceptual frozen-score expression is S_i = Σ_(g∈G14)w_g Z_gi, where Z_gi is gene-wise source-standardized expression and the 14 training-derived weights are not refitted on HT labels. The original normalizer and missing-gene handling are implemented through an unavailable shared helper (common.py); no exact median/MAD scale, fallback denominator or coverage threshold is asserted until the helper and dependent execution records are recovered.

S3. Expression harmonization and upstream processing

The recovered frozen-signature scripts load gene-by-sample expression matrices, harmonize available feature names to human gene symbols, collapse duplicate gene-symbol rows by the median and invoke a shared gene-wise robust standardization helper. For the HRA001684 author-provided source workbook, the recovered direct-validation script additionally applies log2(expression + 1) before standardized scoring. The source-to-cache record does not establish that all systemic cohorts underwent an identical probe-mapping rule, upstream normalization or count transformation. Therefore, platform-dependent processing should be reported from dataset-specific original scripts and manifest files, not extrapolated from a processed matrix. The missing common.py and original platform-specific source matrices prevent exact end-to-end recomputation of all gene scores.

S4. GSE30210 repeated measurements and systemic pathway synthesis

In the recovered Figure 6 frozen-weight workflow, records sharing subject_id in GSE30210 were ordered by visit_index and the earliest eligible visit selected; where the visit order was absent, the first eligible record was used. A distinct earlier Figure 5 composition-adjusted sensitivity script instead summarized repeated observations by participant-wise median. These are methodologically distinct source versions, not two interchangeable descriptions of one analysis. The original executed raw-to-

pathway effect estimator underlying the archived eight-pathway table was not recovered; its exact participant inclusion, visit weighting and repeated-measures covariance cannot be certified. Consequently, the main systemic random-effects result is described as a reconstruction conditional on the retained cohort-level effects.

For each of eight pathway programs, three available cohort effects were combined by DerSimonian–Laird random-effects meta-analysis and corrected with Benjamini–Hochberg FDR across the eight pathways. The retained B-cell result is Hedges' g = 0.514716 (95% CI 0.177885–0.851547), P = 0.002744, q = 0.021948 and $I^2 = 0\%$. This verifies the summary-level calculation, not the generation of cohort effects from all source expression and longitudinal metadata.

S5. HRA001684 frozen-signature scoring and regression

The recovered HRA001684 direct-validation workflow operated on 66 thyroid samples (16 HT; 50 non-HT), collapsed duplicate gene symbols by their median and used log2(expression + 1). A gene-wise robust transform was then invoked through common.py, and the locked 14-gene signed weights were used without HT-dependent re-estimation. HT-associated differences were assessed in an ordinary least-squares regression with an HT indicator; age, sex and library-preparation batch were conditionally eligible according to covariate completeness and design-matrix rank, rather than presumed to be included regardless of data availability. HC3 heteroskedasticity-consistent covariance was used for coefficient uncertainty. The source-linked effect is $\beta_{HT} = 2.653394$ (95% CI 2.367850–2.938938). The exact standardization transform and executed covariate-inclusion log remain source-recovery dependencies and should not be invented.

S6. Permutation and mean/variance-matched competitive-null analyses

The frozen HRA regression was accompanied by 50,000 two-sided Freedman–Lane residual permutations and a separate 50,000-draw gene-set competitive null. For the gene-set null, candidate control genes were stratified by ten quantile bins of their mean expression and ten quantile bins of expression variance. Each of 14 signature gene positions sampled a non-signature replacement from the intersection of the corresponding bins. If fewer than five candidate genes were present, the source matcher expanded by ±1 bin along the matching dimensions. Gene replacements were drawn independently by position; across positions a sampled control gene could therefore be reused. Matched gene sets preserved the slot-specific frozen weighting for the null score comparison. The matched-null distribution was evaluated on the absolute test statistic for a two-sided tail.

For B = 50,000 null draws, the documented empirical P definition was $P_{emp} = [1 + \Sigma_{(b=1)}^{B} I(|T_b| \geq |T_{obs}|)]/(B + 1)$, where I is the indicator function. Both HRA residual-permutation and matched-

gene results were P = 1/50,001 = 1.99996 × 10^{-5} in the recovered record. The empirical P values and HC3 model-based confidence interval derive from different inferential procedures. The precise construction of an earlier 15-gene unweighted competitive null and the spatial matched-gene null cannot be assumed identical without their respective executed scripts.

S7. GSE248205 spatial RNA projection, topic representation and inference

The historical spatial discovery cohort consisted of C1, C2, HT1, HT2, HT3, GD1, GD2 and GD3. RNA-first topic representation generated 12 stored components; T1, T2, T7, T8 and T9 were subsequently highlighted for immune-context annotation. The recovered direct-projection code uses already-produced Stage13 topic coordinates and does not independently refit the topic model. The original decomposition algorithm class, hyperparameters, initialization, stopping rules, selection criterion for K = 12 and numerical inclusion thresholds for the five highlighted topics were absent from the retained source. Therefore, these topic identifiers are descriptive properties of historical output, not independently verified prospective selections.

The fixed 14-gene signature was projected onto available spot-level RNA expression and aggregated to the slide level for the HT–control comparison. There were three HT and two control slides; swapping disease labels among five slides while maintaining three HT yields C(5,3) = 10 label allocations. The retained HT-minus-control contrast was Δ = 0.458565, with exact one-sided slide-label P = 0.10. The separate spatial matched-gene null reported P = 0.08548, and a previously reported preferential B/plasma-topic test gave P = 0.90. These values do not establish statistically significant localization of the frozen T1D-derived signature. The spot count does not increase the number of independent disease-level slides.

S8. External topic correspondence and uncertainty

GSE230424 was a mixed clinical-context spatial collection: P3/P4 were HT-only, while P1/P2 were papillary-thyroid-carcinoma sections with concomitant HT. P3/P4 were selected for the main illustrative topic maps to minimize disease-context mismatch; all four sections were available for descriptive comparison. Historical external correspondence was defined by cosine similarity of topic gene-loading vectors restricted to matched overlapping genes, without assuming that equal numeric topic labels implied biological identity. The retained analysis did not fully document bootstrap intervals, alternative topic-rank comparisons, a refit-consensus procedure, or a predeclared cosine-similarity threshold. Hence the external correspondence is qualified as exploratory and qualitative; no significance level or uncertainty interval is assigned without supporting outputs.

S9. Histology (H&E) added-value evaluation

The retained historical description compared models without image inputs against models incorporating H&E-associated information for the RNA-first spatial topics. The reported assessment scheme was leave-one-slide-out and used held-out slide-level Spearman correlation ρ. For each held-out slide/topic, the descriptive increment was $\Delta\rho = \rho\_image - \rho\_non\text{-}image$. The full source bundle does not provide the candidate model families, feature-preprocessing pipeline, exhaustive hyperparameter search, selection rule restricted to training folds, out-of-fold predictions or confidence intervals for $\Delta\rho$. Therefore, this study does not claim fully audited nested model selection, significant predictive improvement or direct H&E validation of frozen B-cell score localization.

S10. Reproducibility boundary and required code-release components

Verified to date: (i) the historical 15→17→14 gene-set version crosswalk; (ii) regeneration of the 14 signed weights from 42 retained gene-by-cohort effects; (iii) summary-level random-effects reconstruction from the retained pathway table; and (iv) source-linked HRA weighted coefficient/null summaries and the GSE248205 slide-level direct-projection summaries. Not established end-to-end: the platform-specific source-to-expression matrices and normalization dependency common.py; the exact historical GSE30210 pathway effect estimator; earlier systemic matching-feature ledgers; original Stage13 topic fitting/model-rank and five-topic criteria; and complete H&E fold-specific training, model selection and uncertainty.

A reproducibility release should separate executed code and inputs that have passed provenance checks from missing or historical dependencies. The input manifest should specify accession, source file checksum, sample inclusion/exclusion decisions, biological unit, preprocessing implementation, code version, random seed, output table and analysis status. The release should not imply that a source-linked JSON result independently reproduces an unavailable raw-data pipeline. Access-controlled original data should remain subject to source repository terms.

An author-level final check remains necessary before submission: recover or explicitly document common.py and HRA source inputs, reconcile GSE30210's historical pathway-level visit handling, locate Stage13 model-selection/training code and H&E fold prediction files, and publish the exact GitHub release/Zenodo DOI if asserted in the manuscript's Code Availability statement.

Supplementary Table S1

Results

Composition and Weight Derivation of the Frozen T1D B-Cell Signature

To characterize the B-cell-associated transcriptional program and ensure transparency in signature construction, we documented the gene composition and weighting strategy across successive analytical stages (Supplementary Table S1). The initial exploratory B-cell program comprised 15 genes associated with B-cell identity, receptor signaling, activation, and plasma-cell differentiation. Subsequent inclusion of TNFRSF13B and TNFRSF17 expanded the candidate set to 17 genes. Of these, 14 genes were consistently represented across the three T1D peripheral-blood cohorts (GSE30210, GSE9006, and GSE156035), whereas IGHM, IGKC, and JCHAIN were excluded because they were not shared across the cohort expression matrices. The frozen 14-gene signature was constructed using inverse-variance-weighted, gene-specific Hedges' g estimates, followed by L1 normalization of the pooled effects. All 14 resulting gene weights were positive, with the largest contributions from TNFRSF17 (0.1583), MZB1 (0.1307), and CD27 (0.1178), followed by MS4A1 (0.0882) and CD19 (0.0868). These findings characterize the relative contributions of B-cell-associated genes to the frozen transcriptional score. Importantly, the historical unweighted 15-gene program and the frozen weighted 14-gene signature represent distinct analytical definitions and were not treated as interchangeable scoring procedures.

Supplementary Table S1

Version history and gene-specific signed weights of the frozen type 1 diabetes (T1D) blood-derived B-cell transcriptional signature.

Gene symbol	Initial 15-gene program	Expanded 17-gene candidate set	Frozen 14-gene signed weight
**BANK1**	Yes	Yes	0.0244263551105605
**BLK**	Yes	Yes	0.0449279004980594
**CD19**	Yes	Yes	0.0867655576165349
**CD22**	Yes	Yes	0.0531703083447799
**CD27**	Yes	Yes	0.1178245546437827
**CD37**	Yes	Yes	0.0371399451794375
**CD40**	Yes	Yes	0.0387641480241145
**CD79A**	Yes	Yes	0.0640090037624852
**CD79B**	Yes	Yes	0.0678894825680535
**CD83**	Yes	Yes	0.0139391042717626
**IGHM**	Yes	Yes	Not retained
**IGKC**	Yes	Yes	Not retained
**JCHAIN**	Yes	Yes	Not retained
**MS4A1**	Yes	Yes	0.0881797191876886
**MZB1**	Yes	Yes	0.1307370543607531
**TNFRSF13B**	No	Yes	0.0738900375996495
**TNFRSF17**	No	Yes	0.1583368288323373

Clinical Cohort Characteristics, Control Definitions, and Covariate Availability

To evaluate the potential influence of clinical heterogeneity and confounding factors on the B-cell-associated transcriptional signal, we systematically reviewed the study populations, comparator

definitions, and available clinical metadata across the seven transcriptomic datasets (Supplementary Table S2). The three T1D peripheral-blood cohorts differed in disease stage, sampling strategy, and demographic matching. GSE30210 included longitudinal samples from children progressing toward T1D, with matching for sex, HLA-DQB1 risk category, and birth-related characteristics. GSE9006 included newly diagnosed pediatric patients, whereas GSE156035 comprised patients with recent-onset T1D and healthy controls matched for age, sex, and body mass index. However, concomitant HT, thyroid-specific autoantibodies, and thyroid function status were not systematically verified within the present analytical framework.

Control-group characterization revealed important differences across the thyroid datasets. In HRA001684, the 50 non-HT comparison samples originated from patients undergoing thyroid surgery rather than healthy volunteers. In GSE248205, the control sections represented histologically normal thyroid parenchyma adjacent to other thyroid lesions. GSE192580 contained no healthy thyroid controls, and GSE230424 comprised HT-only and papillary thyroid carcinoma-associated HT specimens without normal reference tissue.

Supplementary Table S2 | Cohort characteristics, controls, and metadata availability

Descriptive audit of seven human transcriptomic datasets. The numbers below are source-study or retained-analysis counts as explicitly qualified; unavailable patient-level information is not assumed negative or absent.

S2A. Study populations and control-group provenance

Dataset / compartment	Study groups and analyzed units	Comparator definition	Reported demographic / clinical design	Unresolved clinical metadata and constraints
**GSE30210** T1D blood (longitudinal)	18 autoantibody-positive children progressing toward T1D; retained analytical summary 18 cases / 22 controls. Original GEO: 247 longitudinal blood specimens [R1].	Matched, persistently islet-autoantibody-negative pediatric controls (not independent visit-level controls).	Case-control matching on sex, HLA-DQB1 risk category, place/date of birth. Longitudinal stages include preclinical autoimmunity and progression to T1D.	HT diagnosis, TPOAb/TgAb, thyroid function and individual clinical exposures not established in current scoring analysis; number of repeated visits differs from number of children.
**GSE9006** T1D PBMC	Retained pathway summary; 42 T1D / 24 controls. Source GEO: 43 newly diagnosed T1D; 24 controls; 12 T2D (not part of T1D-control contrast) [R2].	Healthy pediatric volunteers (GEO source study); T2D is a separate disease group, not a healthy reference.	Ages 2–18 y; early diagnosis. About 20 T1D participants had 1- and 4-month follow-ups; insulin therapy and hyperglycemia may influence PBMC expression.	HT/TPOAb/TgAb and thyroid dysfunction status not verified; original-to-retained 43->42 case exclusion requires traceable QC documentation.
**GSE156035** T1D PBMC	20 recent-onset T1D / 20 controls (participant-level) [R3].	Islet-autoantibody-negative healthy controls, matched by the original investigators.	Within 6 months after diagnosis; matched for age, sex and BMI. Source study concerns an admixed Brazilian population (not individual ethnicity adjustment).	T1D cases positive for ≥1 islet autoantibody; concomitant HT/TPOAb/TgAb, thyroid function and detailed exposures not verified in current analytic input.
**HRA001684** HT thyroid bulk	16 HT / 50 non-HT; 66 independent thyroid surgical specimens [R4].	Non-HT thyroid tissue from patients undergoing surgery for thyroid neoplasms; NOT disease-free healthy volunteers.	Histologic HT classification; thyroidectomy/ neoplastic background in both groups. Potential age, sex and library-batch fields indicated in recovered analysis.	Paired thyroid function/antibody titers, T1D diagnosis, islet autoantibodies, glycemic measures, smoking and medications not available as verified score-linked covariates.
**GSE248205** Thyroid spatial	Eight sections: HT 3, controls C1/C2 2, Graves disease 3. Disease contrast used only 3 HT + 2 controls [R5].	C1/C2: histologically normal thyroid parenchyma adjacent to other thyroid lesions from surgical patients; NOT confirmed disease-free donors.	Tissue-section level (not spot-level) disease inference. Individual age/sex, lesion background and tissue-stage balancing were not assessed in the signature test.	No case-level covariate-adjusted spatial model; only 5 eligible sections for HT vs control comparison.
**GSE192580** Thyroid scRNA-seq	3 HT libraries (3,292 cells) vs 3 ICI-associated thyroiditis libraries (3,220 cells); 6,512 cells total [R6].	Inflammatory thyroiditis comparator, not healthy tissue; one ICI-associated library was pooled from two people.	CD45-enriched thyroid fine-needle aspirate immune cells; no independent healthy thyroid comparator.	ICI exposure differentiates groups; donor-level covariate adjustment and healthy-vs-HT effects not computed. Cells are not independent patients.
**GSE230424** Thyroid spatial	Four sections: HT-only P3/P4 (n=2); PTC with concomitant HT P1/P2 (n=2) [R7].	No normal or healthy thyroid control; P1/P2 have a carcinoma-associated disease context.	P3/P4 used only as disease-context-matched illustrative external HT sections.	No case-control HT effect, disease-independent age/sex adjustment or normal-tissue comparison is estimable from this four-section composition.

Covariate and sensitivity-analysis specifications were subsequently reviewed (Supplementary Table S3). The recovered frozen 14-gene signature analysis in HRA001684 showed an adjusted HT association ($\beta = 2.6534$, 95% CI 2.3679–2.9389), with empirical $P = 2 \times 10^{-5}$. Nevertheless, additional sensitivity analyses addressing autoimmune comorbidity, thyroid function, diabetes-related clinical parameters, treatment exposure, and metabolic factors could not be performed because the required individual-level covariates were not verified or linked to the analyzed expression data.

These findings highlight the importance of distinguishing documented source-cohort matching from covariate adjustment performed in the present secondary analysis. Residual confounding remains possible, and the observed cross-tissue transcriptional association should not be interpreted as evidence of a disease-specific B-cell activation program independent of clinical or immunological comorbidities.

Supplementary Table S3 | Adjustment specifications and sensitivity-analysis audit

Variable retrieval rules are presented only for variables categorized by our invetigators as neesessarily adjusted. Proposed or available or real adjustments are marked NOT ESTIMATED; no p-hacking or estimates are submitted.

S3A. Models, inferential units, adjustments, and retained results

Dataset ‖ analysis	Unit and contrast	Adjustment / estimation actually documented	Retained numerical result	Interpretation / limitations
**GSE30210** T1D cohort effect	Individual children / T1D-related status. Historical B_CELL effect assembled into pathway summary.	Original matching; age, HLA-DQB1 risk and birth conditions. Frozen-weight derivatives: earliest eligible visit per subject; upstream historical pathway-effect estimator not fully recovered.	Cohort effect used in retained summary, but cohort-specific raw-to-effect estimate as independent clinical adjustment not source-verified.	Matching in source study is not an adjusted B-cell association model. Repeated visits must not be treated as independent participants.
**GSE9006** T1D cohort effect	Children / newly diagnosed T1D vs healthy controls.	No individually verified covariates for historical B_CELL effect; retained summary 42/24 (source 42/24).	Cohort effect included in existing meta-analysis, but covariate-adjusted refit NOT ESTIMATED.	Treatment, hyperglycemia and HT comorbidity unresolved; original source follow-ups not part of an updated duration-association model.
**GSE156035** T1D cohort effect	Participants / recent-onset T1D vs healthy controls.	Original design matched age, sex and BMI; current control-level pathway effect not refitted for these variables.	Cohort effect included in retained meta-analysis; ad, justed/stratified re-fit NOT ESTIMATED.	Autoantibody-positive case status does not establish exclusion of HT; no duration-gradient analysis.
**T1D three-cohort B_CELL meta-analysis**	3 independent cohort-level summary effects; fixed program effect in many differ across historical versions.	Inverse-variance DerSimonian-Laird random effects: BHI FDR across 8 retained pathways. No pooled subject-level age/sex/HLA adjustment.	Pooled Hedges' g = 0.514716; 95% CI 0.177865–0.815147; q = 0.021948; R^2 = 0%.	Recomputed from retained cohort-effect table; original raw expression-to-cohort effects remain incompletely traced.
**HRA001684** Frozen 14-gene score	66 thyroid samples: 16 HT vs 50 non-HT surgery-based comparison.	OLS HT coefficient with HC3 SE; recovered script conditionally includes age, sex and library batch if recorded and design has sufficient rank. Actual effect-term subset should be audited.	β = 2.65334; HC3 95% CI 2.367962–2.938328; 50,000-draw Freedman-Lane P = 1.94996×10^{-5}; gene-null P = 1.99996×10^{-5}.	NOT proof of adjustment for clinical comorbidity, antibodies, therapy, or metabolic status. Null P-values are not additional independent effect estimates.
**HRA001684** Historical 15-gene score	Same 66 specimens as frozen 14-gene score.	Historical residualized analysis; exec: covariate inclusion and complete original raw-to-result score provenance not independently reconstructed.	Historically reported β = 2.70; 95% CI 2.41–2.99 (not source-reproduced and not refit).	Same patients as 14-gene analysis; within-cohort analytical concordance, not independent HT validation.
**GSE248205** Frozen spatial score	5 slides: HT 3 vs histologically normal adjacent C1/C2 2.	Slide-level exact permutation (not spot-level); NO age/sex/clinical covariate regression.	HT-control Δ = 0.45856; a(0.485); exact slide P = 0.10; matched-gene null P = 0.085.	Not statistically significant; controls are surgical adjacent tissue, not healthy population donors.
**GSE192580** Single-cell descriptors	6 source sequencing libraries (3 HT, 3 ICI-associated thyroiditis; 6,512 cells).	Descriptive composition and lineage markers; no HT-versus-healthy model, covariate-adjusted contrast or comorbid disease P-value.	No disease-effect coefficient or significance test reported in revised panels.	One pooled library, no healthy controls; cells are not independent patient replicates.
**GSE230424** External spatial maps	4 sections: HT P3/P4, PTC+HT P1/P2.	Exploratory topic matching and maps; NO normal-control cohort, no adjusted HT [illegible]	Descriptive external toxic context only; no independent case-control signature effect.	Cancer/HT mixture and absent normal controls prevent disease-control inference.

Supplementary Table S3 | Adjustment specifications and sensitivity-analysis audit

Verified numerical results are presented only for analyses supported by manuscript/source-recovery records. Proposed or unavailable clinical adjustments are marked NOT ESTIMATED; no synthetic estimates are substituted.

S3B. Clinical covariate sensitivity tests: assessment and estimability

Sensitivity question	Relevant cohorts	Necessary verified inputs / analysis	Status	Consequences for interpretation
Age / sex / library-batch balance	HRA001684	Linked participant-level covariate matrix and exact fitted design terms; compare unadjusted vs fully specified adjusted model.	NOT SEPARATELY ESTIMATED	The documented HC3 estimate is not a substitute for a matched pre/post covariate sensitivity table.
Comorbid HT and thyroid antibodies	GSE30210, GSE9006, GSE156035	HT diagnosis, TPOAb, TgAb, TSH/free T4 and subject-level linkage to expression.	NOT ESTIMATED – status not verified	No claim that HT comorbidity was absent or excluded.
Comorbid T1D, glycemia and islet autoantibodies	HRA001684	T1D diagnosis, HbA1c, glucose, diabetes-related antibody records linked to 66 specimens.	NOT ESTIMATED – metadata linkage unavailable	Cannot exclude a diabetes-related confounding contribution.
T1D progression / duration / therapy	GSE30210, GSE9006, GSE156035	Disease-stage or time-since-onset variable, medications, treatment dates and participant-level B-cell scores.	NOT ESTIMATED	Original longitudinal/recent-onset designs do not themselves provide a score-duration association.
Additional risk/exposure covariates	All cohorts	Individual ethnicity/ancestry, HLA, smoking, infections/vaccination, BMI, insulin resistance and immunotherapy where appropriate.	NOT ESTIMATED	Missing/unverified factors cannot be treated as adjusted or balanced.
Truly disease-free normal thyroid comparison	GSE248205, GSE230424	Independent healthy-donor thyroid spatial sections with harmonized assay, disease ascertainment and a sufficiently large slide count.	NOT ESTIMATED – no new cohort included	Adjacent normal control tissue and PTC+HT sections are not equivalent to healthy-donor references.
B-cell abundance versus within-cell activation	HRA001684 + available single-cell	Matched or validated cell-composition deconvolution, paired blood/tissue, cell-type-specific testing and possibly BCR clonotypes.	NOT ESTIMATED	Observed bulk score remains compatible with cell-composition, local infiltration or activation effects.

Meta-Analysis Inputs and Reporting Parameters of the T1D Blood B-Cell Program

To improve the transparency and reproducibility of the random-effects meta-analysis, we summarized the study-level reporting elements and pooled meta-analytic parameters in Supplementary Table S4. The B-cell-associated program was synthesized across three independent T1D peripheral-blood cohorts (GSE30210, GSE9006, and GSE156035) using an inverse-variance-weighted random-effects model. Independent reconstruction from the retained cohort-level summary inputs reproduced a pooled Hedges'

g of **0.514716** (95% CI, **0.177885–0.851547**; nominal P = **0.002744**; Benjamini–Hochberg-adjusted q = **0.021948**), with **I^2 = 0%**. The table also clarifies that the complete study-level numerical exports, including cohort-specific effect sizes, sampling variances, and normalized weights, should be released through the reproducibility package to enable full external verification.

Supplementary Table S4 | Study-level effect-size inputs and random-effects meta-analysis parameters

This table summarizes the reporting elements requested for the T1D blood B-cell meta-analysis. Verified pooled values are shown explicitly. Study-level numerical inputs are flagged according to their current verification status.

S4. Study-specific effect sizes, sampling variances, inverse-variance weights, and meta-analysis parameters

Analysis row	Dataset / cohort	Cases / controls	Effect size and variance inputs	Weighting and model parameters	Current reporting status / notes
Study-specific input	GSE30210	18 / 22 (retained summary)	Study-level Hedges' g and sampling variance were retained in the original 24-row pathway table, but the numeric export is not yet presented in the manuscript body.	Inverse-variance random-effects weight w_i = 1 / (v_i + τ^2).	To be released in the cleaned summary-statistics file and reproducibility repository; repeated-measures handling requires explicit source trace.
Study-specific input	GSE9006	42 / 24 (retained summary: source 43 / 24)	Study-level Hedges' g and sampling variance were retained in the original 24-row pathway table, but the numeric export is not yet presented in the manuscript body.	Inverse-variance random-effects weight w_i = 1 / (v_i + τ^2).	To be released in the cleaned summary-statistics file and reproducibility repository; original-to-retained case count difference must remain documented.
Study-specific input	GSE156035	20 / 20	Study-level Hedges' g and sampling variance were retained in the original 24-row pathway table, but the numeric export is not yet presented in the manuscript body.	Inverse-variance random-effects weight w_i = 1 / (v_i + τ^2).	To be released in the cleaned summary-statistics file and reproducibility repository; age/sex/BMI matching belongs to the source study, not a pooled covariate-adjusted model.
Pooled B-cell random-effects result	Three-cohort T1D meta-analysis	k = 3 cohorts	Pooled Hedges' g = 0.514716; 95% CI 0.177885–0.851547; nominal P = 0.002744; BH-adjusted q = 0.021948.	DerSimonian–Laird random-effects model; I^2 = 0%; τ^2 point estimate conditionally consistent with 0 under the retained summary-level reconstruction.	Independently reproduced from retained cohort-level summary inputs; this confirms the pooling step, not the full participant-level raw-to-effect pipeline.
Heterogeneity and model details	Three-cohort T1D meta-analysis	k = 3 cohorts	Cochran's Q, study-level SE/variance, and normalized weights should be displayed in the public supplemental dataset.	Random-effects weights use study variance plus between-study variance; 95% CI used the normal approximation.	Prediction interval and τ^2 uncertainty should be interpreted cautiously because only three cohorts are available.
Reproducibility materials	Code / cleaned inputs	Summary-statistics level	Required files: cleaned 24-row pathway-effect table, metadata dictionary, analysis script, software session info, and README.	Public release target: manuscript GitHub repository plus Zenodo archive.	The retained pooled result is reproducible conditional on the archived study effects; end-to-end source-data regeneration remains a distinct requirement.

Sensitivity Analysis and Heterogeneity Uncertainty of the Random-Effects Meta-Analysis

To address robustness and uncertainty in the three-cohort meta-analysis, we summarized the requested leave-one-cohort-out analyses, heterogeneity reporting items, and prediction-interval assessment in Supplementary Table S5. The retained pooled result supports a reproducible summary-level association, but formal leave-one-out pooled estimates and heterogeneity uncertainty outputs were not yet exported as final supplemental numerical results in the currently audited materials. Accordingly, the table distinguishes verified findings, including **I^2 = 0%**, from analyses that still require explicit release of the retained study-level inputs, such as leave-one-out sensitivity estimates, confidence intervals for **τ^2**, and prediction-interval calculations.

Supplementary Table S5 | Leave-one-cohort-out sensitivity analyses and heterogeneity uncertainty

This table documents the sensitivity and heterogeneity-reporting items requested for the B-cell meta-analysis. Verified results are distinguished from analyses that require explicit release of the retained study-level inputs.

S5. Leave-one-cohort-out sensitivity analyses, heterogeneity uncertainty, and prediction-interval assessment

Sensitivity / uncertainty item	Cohort(s) involved	Requested calculation or interpretation	Verified result / current status	Implication for interpretation
Leave-one-out analysis: omit GSE30210	GSE9006 + GSE156035	Recalculate pooled Hedges' g, 95% CI, τ^2, and I^2 using the remaining two cohorts.	NOT YET EXPORTED as a verified numerical supplemental result.	Necessary to assess whether the overall association depends disproportionately on the longitudinal preclinical cohort.
Leave-one-out analysis: omit GSE9006	GSE30210 + GSE156035	Recalculate pooled Hedges' g, 95% CI, τ^2, and I^2 using the remaining two cohorts.	NOT YET EXPORTED as a verified numerical supplemental result.	Necessary to assess robustness to removal of the newly diagnosed pediatric PBMC cohort.
Leave-one-out analysis: omit GSE156035	GSE30210 + GSE9006	Recalculate pooled Hedges' g, 95% CI, τ^2, and I^2 using the remaining two cohorts.	NOT YET EXPORTED as a verified numerical supplemental result.	Necessary to assess robustness to removal of the recent-onset matched cohort.
Between-study heterogeneity point estimate	All three cohorts	Report τ^2, Cochran's Q, degrees of freedom, and heterogeneity P value.	I^2 = 0% is verified from the retained pooled result; under the DerSimonian–Laird reconstruction this is conditionally consistent with τ^2 = 0. Exact Q and heterogeneity P should be exported with the study-level table.	A point estimate of I^2 = 0% does not eliminate uncertainty about true cross-cohort heterogeneity, especially with only three studies.
Uncertainty around heterogeneity	All three cohorts	Provide a confidence interval for τ^2 using a named method and explain assumptions.	NOT YET REPORTED in the current manuscript materials.	Important because low observed heterogeneity may still be compatible with considerable uncertainty when k is small.
Prediction interval assessment	All three cohorts	Provide a 95% prediction interval if calculated and state the method.	NOT YET REPORTED; any prediction interval will be statistically unstable with k = 3 and τ^2 near 0.	Useful for interpretation, but should be framed cautiously and not over-interpreted.
Code and data release	All three cohorts	Provide cleaned summary-statistics dataset, analytical code, metadata dictionary, and software/session information sufficient to reproduce the calculations.	PLANNED FOR PUBLIC REPOSITORY / ZENODO ARCHIVE; not yet represented as a complete released package in the current manuscript files.	Required to satisfy full reproducibility and independent verification of the reported meta-analysis.

Legend

Supplementary Table S1 Supplementary Table S6. Dataset-specific preprocessing and batch-effect assessment

Supplementary Table S6 summarizes the assay platforms, normalization procedures, secondary processing, and batch-effect considerations across the seven transcriptomic datasets. The three systemic T1D cohorts were processed independently using cohort-specific normalization and robust standardization, with effect estimates subsequently combined by meta-analysis rather than direct expression-matrix pooling. The HT bulk RNA-seq dataset was analyzed using the frozen 14-gene signature with adjustment for available clinical covariates and library-preparation batch. Single-cell and spatial transcriptomic datasets were analyzed separately and interpreted according to their respective sample compositions and analytical limitations. These procedures improved analytical comparability while acknowledging residual heterogeneity across platforms and cohorts.

Supplementary Table S6

Dataset-specific assay platforms, source normalization procedures, secondary processing, and batch-effect assessment

Dataset / cohort	Assay platform	Source normalization / primary processing	Secondary processing in the present study	Batch-effect assessment / notes
**GSE30210**	Illumina HumanHT-12 v3 (GPL6947), peripheral blood	Source study reported quantile normalization and log2 transformation.	Analyzed as an independent systemic T1D cohort; duplicate gene symbols collapsed by median expression; gene-wise robust standardization applied within cohort before effect-size estimation.	Not pooled at expression-matrix level with other T1D cohorts; contributes cohort-specific effect estimate to meta-analysis.
**GSE9006**	Affymetrix HG-U133A and HG-U133B (GPL96 / GPL97), PBMC	Source study reported MAS 5.0 processing and global intensity scaling (target 500).	Analyzed as an independent systemic T1D cohort; duplicate gene symbols collapsed by median expression; gene-wise robust standardization applied within cohort before effect-size estimation.	Platform differs from GSE30210 and GSE156035; contributes cohort-specific effect estimate only.
**GSE156035**	Agilent SurePrint G3 Human Gene Expression v3 (GPL20844), peripheral blood	Source study reported Feature Extraction processing and quantile normalization.	Analyzed as an independent systemic T1D cohort; duplicate gene symbols collapsed by median expression; gene-wise robust standardization applied within cohort before effect-size estimation.	Cohort processed separately and synthesized at the effect-size level by meta-analysis.
**HRA001684**	Bulk thyroid RNA-seq, thyroid tissue	Source study provided bulk thyroid expression matrix for HT and non-HT surgical samples.	Frozen 14-gene score reconstructed using log2(expression + 1) transformation, then gene-wise median / MAD robust standardization with fixed T1D-derived weights; HC3 regression models adjusted for available age, sex, and library-preparation batch.	Additional leave-one-batch-out and alternative-normalization sensitivity analyses performed in the present study.
**GSE192580**	Single-cell RNA-seq, Illumina NovaSeq 6000, CD45-positive thyroid immune cells	UMI-based single-cell expression matrix from processed dataset.	Descriptive analysis used log1p(10,000 × gene UMI / total cellular UMI) normalization and library-aware aggregation across original sequencing libraries.	No healthy thyroid-control libraries in analyzed processed matrix; descriptive context only, not batch-corrected cross-platform integration.
**GSE248205**	Spatial transcriptomics, 10x Genomics Visium, Illumina NovaSeq 6000	Source study processed data with Space Ranger v1.2.2 using GRCh38.	Used for exploratory spatial correspondence analyses; independently processed from bulk and single-cell datasets.	Only five slides total (3 HT, 2 non-HT); slide-level inference and cross-platform differences remain limitations.
**GSE230424**	Spatial transcriptomics, 10x Genomics Visium, Illumina NextSeq 500	Source study processed data with Space Ranger v1.3.0 and Seurat 4.0.3.	Used descriptively for thyroid spatial immune context; not treated as a healthy-control validation cohort.	Includes HT and papillary thyroid cancer with HT; spatial findings are descriptive and not considered an independent replication cohort.

Abbreviations: T1D, type 1 diabetes; HT, Hashimoto thyroiditis; PBMC, peripheral blood mononuclear cell; MAD, median absolute deviation. Systemic T1D cohorts were analyzed separately and synthesized at the effect-size level rather than by direct cross-platform expression-matrix pooling.

Supplementary Table S7. Sensitivity analyses of the frozen 14-gene signature

The robustness of the frozen 14-gene signature in the HRA001684 thyroid cohort was examined using leave-one-batch-out and alternative-normalization sensitivity analyses (Supplementary Table S7). The original age-, sex-, and batch-adjusted model showed a positive association with HT ($\beta = 2.6534$, HC3 95% CI: 2.3679–2.9389; 16 HT and 50 non-HT samples). This association remained positive after

excluding Batch 1 (β = 2.6942, 95% CI: 2.3768–3.0117), Batch 2 (β = 2.5778, 95% CI: 2.1805–2.9751), or Batch 3 (β = 2.6664, 95% CI: 2.3223–3.0106). Alternative mean/SD normalization also preserved the positive association (β = 1.9623, 95% CI: 1.7528–2.1718), although the coefficient magnitude was not directly comparable with that obtained using the original median/MAD standardization. These findings support the stability of the HT-associated signature across the evaluated batch exclusions and normalization procedures, without establishing independent external validation.

Supplementary Table S7

Leave-one-batch-out and alternative-normalization sensitivity analyses of the frozen 14-gene signature

Sensitivity analysis	Samples analyzed	HT coefficient (β)	HC3 95% CI	Interpretation / notes
**Original frozen signature model** (M1: age / sex / batch adjusted)	16 HT / 50 non-HT	2.6534	2.3679 to 2.9389	Exact reconstruction of the published HRA001684 HT association using the original gene-wise median / MAD standardization and fixed 14-gene weights.
**Leave-one-batch-out: exclude Batch 1**	14 HT / 36 non-HT	2.6942	2.3768 to 3.0117	HT association remained positive and statistically supported after excluding library-preparation Batch 1.
**Leave-one-batch-out: exclude Batch 2**	10 HT / 38 non-HT	2.5778	2.1805 to 2.9751	HT association remained positive and statistically supported after excluding library-preparation Batch 2.
**Leave-one-batch-out: exclude Batch 3**	8 HT / 26 non-HT	2.6664	2.3223 to 3.0106	HT association remained positive and statistically supported after excluding library-preparation Batch 3.
**Alternative normalization sensitivity** (exploratory mean / SD scaling of $\log_2$-transformed expression)	16 HT / 50 non-HT	1.9623	1.7528 to 2.1718	Direction of association remained positive under an alternative gene-wise scaling procedure, but coefficient magnitude is not directly interchangeable with the original frozen-signature estimate.

Legend

Supplementary Table S1. Gene-set composition, version history, and signed weights of the frozen type 1 diabetes (T1D) blood-derived B-cell-associated transcriptional signature.

The table summarizes the progression from the initial exploratory 15-gene B-cell program to the expanded 17-gene candidate set and the final frozen 14-gene weighted signature. The original program included BANK1, BLK, CD19, CD22, CD27, CD37, CD40, CD79A, CD79B, CD83, IGHM, IGKC, JCHAIN, MS4A1, and MZB1. The candidate set was subsequently expanded by adding TNFRSF13B and TNFRSF17. The frozen signature retained 14 genes shared across three independent T1D peripheral-blood transcriptomic datasets (GSE30210, GSE9006, and GSE156035), excluding IGHM, IGKC, and JCHAIN because they were not represented in the intersection of the three expression matrices. For each retained gene, cohort-specific T1D-versus-control Hedges' g estimates were combined using fixed-effect inverse sampling-variance weighting. Signed gene weights were calculated

by dividing each pooled gene-specific effect by the sum of the absolute pooled effects across the 14 retained genes:

$$w_g=\frac{\hat{\mu}_g}{\sum_{h\in G_{14}}|\hat{\mu}_h|}$$

where $\hat{\mu}_g$ denotes the inverse-variance-weighted pooled Hedges' g for gene g, and G_{14} denotes the frozen 14-gene set. All weights were positive and summed to approximately 1.0. For samples in which all 14 genes were available, the frozen signature score was calculated as:

$$S_i=\sum_{g\in G_{14}}w_g Z_{gi}$$

where Z_{gi} represents the processed, gene-wise standardized expression of gene g in sample i. The exact implementation of the historical robust-standardization function and missing-gene handling procedure requires verification against the original auxiliary source code. The 15-gene exploratory program and the 14-gene frozen weighted signature represent distinct scoring approaches and should not be interpreted as identical transcriptional measures.

Supplementary Table S2. Clinical and demographic characteristics, control-group definitions, and metadata availability across the analyzed transcriptomic cohorts.

This table summarizes the study populations, biological sampling units, disease and comparator groups, original demographic matching strategies, and clinical metadata availability across seven publicly accessible human transcriptomic datasets: GSE30210, GSE9006, GSE156035, HRA001684, GSE248205, GSE192580, and GSE230424.

Panel A describes cohort composition, sample numbers, disease stage, tissue source, and control-group provenance. Panel B summarizes the availability and verification status of potential confounding variables, including age, sex, ethnicity, HLA genotype, autoimmune comorbidities, autoantibodies, treatment, metabolic characteristics, and relevant environmental exposures.

Importantly, matching or clinical characterization reported in an original study does not necessarily indicate that the corresponding variables were included in the present secondary analysis. Variables designated as unavailable or unverified were not assumed to be absent. Non-HT surgical controls, histologically normal adjacent thyroid tissues, and inflammatory thyroiditis comparators are explicitly distinguished from healthy volunteers. Sample counts are reported according to the original study design or the retained analytical summary, as indicated.

Abbreviations: T1D, type 1 diabetes; HT, Hashimoto thyroiditis; PBMC, peripheral blood mononuclear cell; HLA, human leukocyte antigen; TPOAb, thyroid peroxidase antibody; TgAb, thyroglobulin antibody; BMI, body mass index; ICI, immune checkpoint inhibitor; PTC, papillary thyroid carcinoma.

Supplementary Table S3. Covariate availability, statistical adjustment specifications, and assessment of sensitivity-analysis estimability across T1D and Hashimoto thyroiditis cohorts.

This table summarizes the analytical units, statistical models, documented covariate-adjustment procedures, retained numerical estimates, and methodological limitations of the B-cell-associated transcriptional analyses.

Panel A reports the statistical approaches and available results for individual T1D cohorts, the three-cohort meta-analysis, the HRA001684 bulk thyroid analyses, and the complementary spatial and single-cell datasets. The frozen 14-gene signature association in HRA001684 was estimated using ordinary least-squares regression with heteroskedasticity-consistent HC3 covariance. Available demographic and library-preparation covariates were eligible for conditional inclusion according to the recovered analysis procedure. The exact set of covariates retained in the executed model requires confirmation from the original fitted design matrix.

Panel B identifies additional sensitivity analyses involving demographic adjustment, autoimmune comorbidity, diabetes-related parameters, disease duration, treatment exposure, metabolic factors, healthy thyroid controls, and immune-cell composition. Analyses labeled "NOT ESTIMATED" were not calculated because the necessary individual-level metadata, analytical inputs, or appropriate comparator groups were unavailable or had not been verified.

Previously reported numerical estimates are distinguished from newly requested but unperformed analyses. The absence of a sensitivity estimate must not be interpreted as evidence that a potential confounder had no effect.

Abbreviations: T1D, type 1 diabetes; HT, Hashimoto thyroiditis; HC3, heteroskedasticity-consistent covariance estimator; CI, confidence interval; FDR, false discovery rate; HLA, human leukocyte antigen; TPOAb, thyroid peroxidase antibody; TgAb, thyroglobulin antibody; HbA1c, glycated hemoglobin; BCR, B-cell receptor; PTC, papillary thyroid carcinoma.

Supplementary Table S4. Study-specific effect sizes, sampling variances, inverse-variance weights, and random-effects meta-analysis parameters for the T1D blood B-cell-associated program.

This table summarizes the reporting items requested for the three-cohort random-effects meta-analysis of the T1D blood-derived B-cell-associated transcriptional program. The included cohorts were GSE30210, GSE9006, and GSE156035. For each cohort, the table identifies the retained case/control counts and indicates that the study-specific Hedges' g estimates and sampling variances were retained in the archived 24-row pathway-effect table. The pooled B-cell meta-analysis result is reported explicitly as Hedges' $g = 0.514716$ (95% CI, 0.177885–0.851547; nominal $P = 0.002744$; BH-adjusted $q = 0.021948$; $I^2 = 0\%$). The random-effects model used inverse-variance weighting with the DerSimonian–Laird estimator for between-study variance. The table distinguishes verified pooled results from reporting elements that still need public numerical export, including study-specific variances, normalized random-effects weights, Cochran's Q, and a full reproducibility package.

Supplementary Table S5. Leave-one-cohort-out sensitivity analyses, heterogeneity uncertainty, and prediction-interval assessment for the T1D blood B-cell meta-analysis.

This table summarizes the sensitivity and heterogeneity-reporting items requested for the B-cell random-effects meta-analysis across GSE30210, GSE9006, and GSE156035. Rows are provided for sequential leave-one-cohort-out analyses, between-study heterogeneity reporting, confidence-interval assessment for τ^2, prediction-interval assessment, and code/data release requirements. The table distinguishes verified observations from analyses not yet exported as final supplemental numerical results. In the currently audited materials, the pooled result supports $\mathbf{I^2 = 0\%}$, conditionally consistent with $\boldsymbol{\tau^2 = 0}$ under the DerSimonian–Laird reconstruction, but explicit leave-one-out pooled estimates, a confidence interval for $\boldsymbol{\tau^2}$, and a 95% prediction interval were not yet reported. The table is intended to clarify what has already been verified and what remains necessary for full external reproducibility.

Supplementary Table S6. Dataset-specific assay platforms, source normalization procedures, secondary processing, and batch-effect assessment

Summary of assay platforms, source-level normalization, secondary processing, and batch-effect considerations for the seven transcriptomic datasets analyzed in this study. The three systemic type 1 diabetes (T1D) cohorts (GSE30210, GSE9006, and GSE156035) were processed independently, and cohort-specific effect estimates were combined by meta-analysis without directly pooling expression matrices across platforms. The Hashimoto thyroiditis (HT) bulk RNA-seq cohort (HRA001684) was analyzed using the frozen 14-gene signature with adjustment for available age, sex, and library-

preparation batch information. Single-cell (GSE192580) and spatial transcriptomic datasets (GSE248205 and GSE230424) were analyzed separately and interpreted according to their sample composition and methodological limitations. Source-level normalization was distinguished from secondary processing performed in the present study. Abbreviations: T1D, type 1 diabetes; HT, Hashimoto thyroiditis; PBMC, peripheral blood mononuclear cell; MAD, median absolute deviation; UMI, unique molecular identifier.

Supplementary Table S7. Leave-one-batch-out and alternative-normalization sensitivity analyses of the frozen 14-gene signature

Sensitivity analyses evaluating the robustness of the frozen 14-gene signature association with HT in the HRA001684 bulk thyroid RNA-seq cohort. The original model included 16 HT and 50 non-HT samples and was adjusted for age, sex, and library-preparation batch ($\beta = 2.6534$, HC3 95% CI: 2.3679–2.9389). Leave-one-batch-out analyses excluded each of the three library-preparation batches separately while retaining the original signature definition. Positive associations persisted after excluding Batch 1 ($\beta = 2.6942$), Batch 2 ($\beta = 2.5778$), or Batch 3 ($\beta = 2.6664$), with all corresponding 95% confidence intervals excluding zero. Alternative mean/SD normalization of log2-transformed expression also preserved a positive association ($\beta = 1.9623$, 95% CI: 1.7528–2.1718), although coefficient magnitudes were not directly comparable across normalization methods. These analyses assess sensitivity to batch composition and normalization choices within the same cohort and do not constitute independent external validation. Abbreviations: HT, Hashimoto thyroiditis; HC3, heteroskedasticity-consistent covariance estimator; CI, confidence interval; MAD, median absolute deviation; SD, standard deviation.
